# Competing ParA Structures Space Bacterial Plasmids Equally over the Nucleoid

**DOI:** 10.1371/journal.pcbi.1004009

**Published:** 2014-12-18

**Authors:** Robert Ietswaart, Florian Szardenings, Kenn Gerdes, Martin Howard

**Affiliations:** 1 Computational and Systems Biology, John Innes Centre, Norwich, United Kingdom; 2 Centre for Bacterial Cell Biology, Newcastle University, Newcastle upon Tyne, United Kingdom; 3 Department of Biology, University of Copenhagen, Copenhagen, Denmark; University of Illinois at Urbana-Champaign, United States of America

## Abstract

Low copy number plasmids in bacteria require segregation for stable inheritance through cell division. This is often achieved by a *parABC* locus, comprising an ATPase ParA, DNA-binding protein ParB and a *parC* region, encoding ParB-binding sites. These minimal components space plasmids equally over the nucleoid, yet the underlying mechanism is not understood. Here we investigate a model where ParA-ATP can dynamically associate to the nucleoid and is hydrolyzed by plasmid-associated ParB, thereby creating nucleoid-bound, self-organizing ParA concentration gradients. We show mathematically that differences between competing ParA concentrations on either side of a plasmid can specify regular plasmid positioning. Such positioning can be achieved regardless of the exact mechanism of plasmid movement, including plasmid diffusion with ParA-mediated immobilization or directed plasmid motion induced by ParB/*parC*-stimulated ParA structure disassembly. However, we find experimentally that *parABC* from *Escherichia coli* plasmid pB171 increases plasmid mobility, inconsistent with diffusion/immobilization. Instead our observations favor directed plasmid motion. Our model predicts less oscillatory ParA dynamics than previously believed, a prediction we verify experimentally. We also show that ParA localization and plasmid positioning depend on the underlying nucleoid morphology, indicating that the chromosomal architecture constrains ParA structure formation. Our directed motion model unifies previously contradictory models for plasmid segregation and provides a robust mechanistic basis for self-organized plasmid spacing that may be widely applicable.

## Author Summary

How DNA is stably inherited through cell division is a fundamental question in cell biology. The most common system that mediates plasmid DNA inheritance in bacteria is through a *parABC* locus, encoding proteins ParA and ParB, and DNA sequence *parC*. These components can position plasmids at equally spaced positions throughout a cell to ensure plasmids are present in both daughter cells when the cell divides into two. Here we study the mechanism by which ParA structures achieve this precise positioning. We show that ParA can direct relatively immobile plasmids over the bacterial chromosome using self-organizing, competitive ParA structures, whose disassembly is induced by plasmid *parC*-bound ParB. More generally these findings will help us to understand transport and regular positioning of intracellular cargo.

## Introduction

*parABC* loci generate equally spaced positioning of many bacterial low copy number plasmids, thereby ensuring stable plasmid inheritance [1]. However, the underlying mechanism of action is not satisfactorily understood. In contrast, plasmid segregation mediated by actin homolog ParM is increasingly well explained and involves filaments that push plasmids apart in a mitotic-like process [2]. Understanding of the *parABC* mechanism is important, as it belongs to the most common class of DNA segregation systems in prokaryotes, used by chromosomes and antibiotic-resistance-carrying plasmids [1], [3]–[5]. Moreover, it is used in other conceptually similar processes, such as chemotactic cluster positioning and partitioning of carbon-fixing carboxysomes [6], [7].

The *parABC* locus present in *Escherichia coli* plasmids such as pB171 and P1 encodes two proteins: ParA, a P-loop ATPase that binds DNA non-specifically in its dimeric ATP-bound form (ParA-ATP for short) [8], [9], and the DNA-binding protein ParB that binds site-specifically to the *parC* region [10], [11]. Fluorescence microscopy has provided evidence for ParA movement over the nucleoid with spatiotemporal oscillations in helix-like structures [12]–[14]. ParB and *parC* are required for these dynamics [12], with ParB promoting the conversion of ParA-ATP to dimeric ParA-ADP (ParA-ADP for short), causing ParA to unbind from the nucleoid [8], [9]. The time period required for nucleoid-disassociated ParA to regain the ability to bind the nucleoid is sufficiently long *in vitro* to ensure that the relative locations of ParA-ADP unbinding and later ParA-ATP rebinding would be uncorrelated due to cytoplasmic ParA diffusion [8]. However, once nucleoid-bound, whether ParA-ATP then polymerizes to form long filaments *in vivo* is currently controversial. Furthermore, the means by which plasmids move under the influence of ParA, and whether ParA polymerization is important for this movement, are also unclear. Nevertheless, the outcome of these ParA dynamics in *E. coli* is equally spaced positioning of plasmid foci over the nucleoid [9], [13]–[15]. This state is achieved regardless of the plasmid focus number n_p_ or cell length, with plasmid foci repositioned in the wake of retracting ParA structures [9].

Several mechanisms have been proposed to explain ParA-mediated plasmid movement. One hypothesis proposes that ParA-ATP polymerizes on the nucleoid to form long filaments and that plasmid translocation is achieved by ParB-stimulated retraction of the polymers, generating effective plasmid-pulling [3], . Other proposals are based on ParA-ATP forming a gradient-like distribution on the nucleoid, without a necessity for polymerization [8], [16]–[21]. It is currently unclear whether any of these mechanisms can explain equal plasmid spacing given the known physiological and biochemical constraints. Here, we therefore investigate which aspects of the polymer and gradient mechanisms are required and sufficient to explain the observed plasmid translocation and equal spacing over the nucleoid.

We begin by showing mathematically that competition between dynamic ParA concentrations on either side of a plasmid can lead to equal plasmid spacing. This mechanism relies on an ability of a plasmid to move towards higher ParA concentrations, but the exact means of such movement is not important. We then investigate theoretically specific means of plasmid movement and examine whether predictions from such models are borne out experimentally. We define a computational diffusion/immobilization model where nucleoid-bound ParA-ATP can anchor diffusing plasmids. We show that diffusion/immobilization can in principle space mobile plasmids equally over the nucleoid. However, experiments measuring increased plasmid mobility in the presence of the pB171 *parABC* locus (*par2*), lead us to disfavor this model. Instead we favour a directed motion mechanism in which ParA structure formation provides directionality to plasmid motion thereby speeding up plasmid movement. The directed motion model produces robust equal plasmid spacing with, on average, relatively symmetric ParA distributions, a prediction we verify experimentally. Furthermore, we show experimentally that ParA organization is dependent on the underlying nucleoid structure, with nucleoid disruption resulting in perturbed plasmid positioning. Our combination of modeling and experiments has for the first time uncovered a robust mechanism for plasmid spacing that unifies previous proposals.

## Results

### ParB-GFP foci are spaced equally over the nucleoid

**Figure 1:**
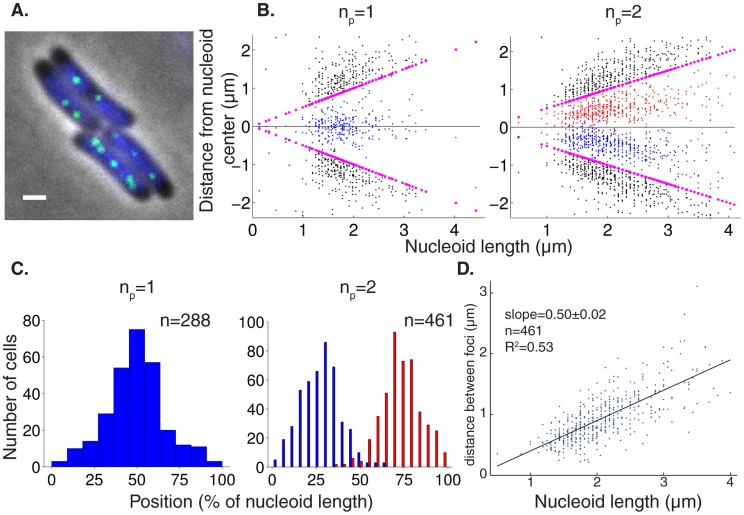
Plasmid foci are equally spaced over the nucleoid. (**A**) Fluorescence localization of plasmid-binding protein ParB-GFP (green) and Hoechst DNA stain (blue) in representative WT *E. coli* cells. Scale bars: 1 µm; plasmid: pFS21 (mini-R1, *parC1^+^*, *parA^+^*, *parB::sfGFP*, *parC2^+^*). (**B**) Scatter plot of plasmid foci positions (blue, red) with respect to nucleoid edges (purple) and cell edges (black) for wild-type cells with n_p_ = 1,2 plasmid foci. (**C**) Histograms of plasmid foci positions shown in (**B**) relative to nucleoid length. (**D**) Scatter plot (blue) of the interplasmid focus distance as a function of nucleoid length in cells exhibiting two plasmid foci. A least square fit (black line) indicates a slope of 0.5.

To study *par2*-mediated plasmid segregation, we investigated ParB-GFP localization, expressed from a *par2-*carrying mini-R1 test plasmid. The *par2* locus containing the *parB::sfGFP* fusion is fully functional as judged by loss-frequency assays (S1A Fig.). As previously described, usage of ParA-GFP and the *tetO-*TetR-mCherry labeling system also does not affect plasmid stability, indicating full functionality [9], [12]. ParB-GFP forms foci that are regularly positioned along the long cell axis *in vivo* (Fig. 1A), consistent with ParB-binding to plasmid-encoded *parC* regions [10], [11]. Since plasmid dynamics occur primarily over the nucleoid, we reasoned that plasmid positioning with respect to the nucleoid rather than cell length is most informative. Therefore we measured ParB-GFP foci localization, together with Hoechst (DNA) stain to determine the nucleoid boundaries. As expected ParB-GFP foci colocalized exclusively with the Hoechst stain, and were equally spaced over the nucleoid (Fig. 1B,C,D for n_p_ = 1,2, S2A,B Fig. for n_p_ = 3,4).

### Mathematical analysis shows that dynamic, competitive ParA concentrations can generate equal plasmid spacing

Several studies have proposed that plasmid positioning is controlled by a concentration gradient of ParA over the nucleoid [8], [16]–[20]. Intuitively in this mechanism, ParB bound to plasmid *parC* (ParB-*parC* complex) interacts with nucleoid associated ParA-ATP, which effectively anchors the plasmid to the nucleoid. At the same time, the ParB-*parC* complex stimulates ParA-ATP hydrolysis causing a local ParA-ATP depletion. These processes could then generate a ParA-ATP gradient which a plasmid is able to follow. Reorganization of ParA gradients under the influence of multiple ParB-*parC* complexes might then lead to equal plasmid spacing. To rigorously understand if, and with what requirements, equal spacing can be achieved we develop here a minimal mathematical model based on the above principles.

We model the nucleoid as a 1d system of length 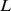 (along the long axis of the cell) on which ParA-ATP and plasmids can interact. Let 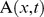 denote the nucleoid-associated ParA-ATP concentration at position 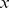 relative to one nucleoid edge at time 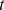. Let 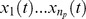 be the positions of the n_p_ plasmids. ParA can bind to the nucleoid with flux 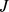. Once bound, ParA-ATP can diffuse along the nucleoid with diffusion constant 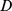. For simplicity, we first assume that the ParA-ATP concentration at each plasmid is zero due to a high ParA-ATP hydrolysis rate. Later on we will relax this assumption. This system can be described by the deterministic reaction-diffusion equations:
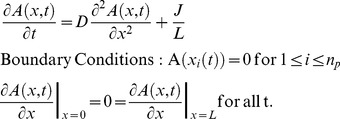



We now use separation of time scales to obtain the steady-state solution for 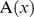: we assume that plasmid motion is much slower than the time for individual ParA-ATP molecules to diffuse over the nucleoid and generate a concentration profile. In this way, the plasmid positions 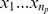 are effectively time-independent and *a priori* unknown. The equation for 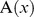 then simplifies to:
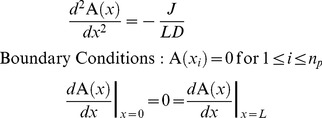
(1)


This equation can be solved by integrating twice using the boundary conditions. The solution is given by:
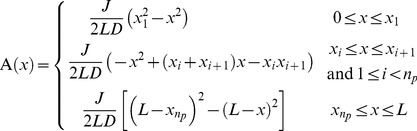
(2)


Next we use these equations to compute the diffusive fluxes of ParA-ATP, 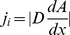, at a plasmid location 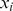, where the + and – superscripts below refer to the flux from the right (+) and left (-) respectively. We find:
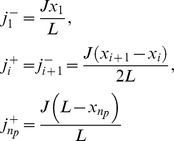



Clearly, a symmetric ParA concentration profile, where fluxes from either side balance, is only possible for 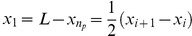. The plasmids are then equally distributed with 
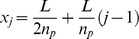
. We note that the predicted inter-plasmid spacing 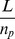 arising from this analysis is consistent with our experimental findings (Fig. 1D, S2B).

Importantly, the above analysis provides insight into the equal spacing mechanism. The key is that the above fluxes depend on the distances either between the plasmid and nucleoid end, or between neighboring plasmids. This feature is a consequence of ParA binding to the nucleoid anywhere, but with ParA release only occurring at a plasmid. In order for these on and off fluxes to balance at steady-state, the off-flux at a plasmid must scale with the inter-plasmid or plasmid-nucleoid-end distance. In this way, non-local information about lengths is converted into local spacing information encoded in the slope of ParA-ATP concentration. For non-equal plasmid spacing, the competing ParA concentrations on either side of a plasmid will be unequal, with one gradient steeper than the other. The steeper gradient corresponds to the side with the greater available space for ParA binding. If a plasmid can preferentially move (on the appropriate slow time scale) towards the side with the locally steepest ParA-ATP concentration, the plasmids are then progressively restored towards equal spacing. As this process occurs, the ParA-ATP concentrations will dynamically reorganize such that a symmetric configuration around a plasmid is reached only when the plasmids are equally spaced. In this state, where the competing ParA-ATP concentrations are symmetric, plasmid movement would no longer have a directional preference and would thus remain, on average, stationary.

So far, we have assumed that the ParA-ATP concentration vanishes at a plasmid, corresponding to very fast ParA-ATP hydrolysis. However, our results also hold true when we only assume that this hydrolysis occurs with a finite rate 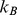, leading to a non-zero concentration of ParA-ATP at a plasmid. This ParA-ATP can then anchor a plasmid to the nucleoid before being hydrolysed. This more general and realistic case is presented in the S1 Text, but our overall conclusions reached above remain unchanged.

From the above analysis, we see that the following conditions are required for equal plasmid spacing: (1) movement of a plasmid towards higher ParA-ATP concentrations. (2) diffusion of (at least a fraction of) ParA-ATP over the nucleoid to ensure formation of competitive concentration gradients. Single molecule tracking experiments *in vitro* support this assumption [17], [18]. (3) ParA-ATP hydrolysis must occur (predominantly) by plasmid-associated ParB-*parC* complexes, again to ensure gradient formation. (4) ParA-ATP must adopt a 1d-like configuration, as previously claimed [9], [13], [14]. If ParA were not organized in this fashion, it would be possible for ParA to diffuse around the sides of a plasmid without encountering the hydrolyzing effect of the ParB-*parC* complex. This would equalize the ParA concentrations on both sides even in the case of asymmetrically placed plasmids, leading to failure of the equal spacing mechanism. This assumption is in line with our subsequent experiments (see below). Due to this proposed 1d-like nature, we will from now on refer to the ParA distributions away from a plasmid as ParA structures. (5) There must be a separation of time scales between plasmid movement and ParA concentration reorganization, as discussed above.

Importantly, this overall mechanism is not reliant on a specific type of plasmid translocation. Any process that would allow a plasmid to move into regions of higher ParA concentration will suffice. In the following sections we therefore analyze different means of plasmid movement and compare them with our experimental data to determine which is used in our *par2* segregation system.

### Diffusion/immobilization model could space highly mobile plasmids equally over the nucleoid

**Figure 2:**
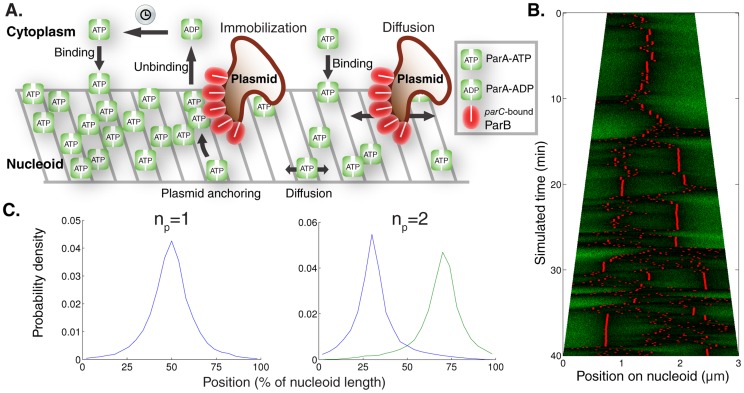
Diffusion/immobilization model can move and maintain plasmids at equally spaced positions. (**A**) Schematic illustration of *par2* diffusion/immobilization model. The clock indicates the slow conversion of cytoplasmic ParA-ADP into cytoplasmic ParA-ATP that is competent to bind to the nucleoid. (**B**) Typical simulation kymograph of diffusion/immobilization model for growing cell, where plasmid (red) diffusion influenced by the local ParA-ATP (green) concentration leads to immobilization initially at mid cell. After plasmid duplication, the system dynamically self-organizes to reacquire equal plasmid spacing. (**C**) Time-averaged plasmid position distributions for diffusion/immobilization model with n_p_ = 1–2 on a simulated nucleoid growing from 1.5 µm to 3 µm in 40 min without plasmid duplication. Plasmid distributions were obtained by sampling positions every 5 s in 36 independent simulations.

In the previous section the mechanistic details of plasmid movement towards a higher ParA concentration were not specified. We now examine a specific implementation involving a diffusion-immobilization mechanism. Using a minimal modelling approach, we assume that nucleoid-associated ParA-ATP can immobilize freely diffusing plasmids through its interaction with the ParB-*parC* complex and that ParA-ATP does not polymerize (Fig. 2A). Since the plasmid will tend to become immobilized in regions of higher ParA-ATP concentration, this process allows for effective plasmid translocation up a ParA-ATP concentration gradient. We also incorporate ParB-*parC*-stimulated ParA-ATP hydrolysis at a plasmid, in accordance with prior experimental data. To further investigate this mechanism, given the known physiological and biochemical constraints, we developed stochastic simulations using a Gillespie algorithm [22]. Here we use standard diffusion for the plasmid movement; below we discuss the potential impact of subdiffusive motion.

In our simulation, a one dimensional lattice with sites of size dx = 5 nm represents the nucleoid. ParA-ATP and plasmids can diffuse on the lattice with diffusion coefficient *D*_A_ and *D*_P_ respectively. Up to 35 ParA-ATP can bind to a plasmid at the same site with reaction parameter k_AB_ reflecting the binding interaction of ParA-ATP and the ParB-*parC* complex [11]. More than one ParA-ATP bound to a plasmid reduces the plasmid diffusion constant to zero. Plasmid-bound ParA-ATP can be hydrolysed with reaction parameter k_B_. Whenever a ParA-ATP hydrolysis event occurs, ParA unbinds from the nucleoid and becomes a cytoplasmic ParA-ADP. ParA-ADP can then be converted into a cytoplasmic ParA-ATP that is competent in DNA binding (cytoplasmic ParA-ATP for short) with a slow reaction parameter k_W_ [8]. Cytoplasmic ParA-ATP can then bind anywhere along the nucleoid with parameter k_on_ (see Materials and Methods and Tables 1,2 for details).

**Table 1:** Reactions and propensities used in the diffusion/immobilization model.

Reactions	Propensities 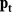
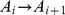 , 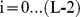	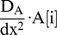
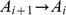 , 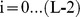	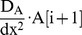
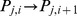 , 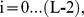 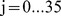	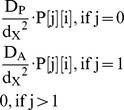
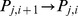 , 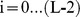 , 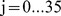	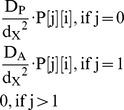
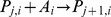 , 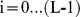 , 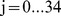	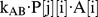
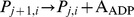 , 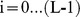 , 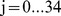	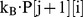
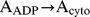	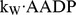
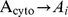 , 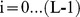	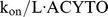

**Table 2:** Parameter values used in the diffusion/immobilization model.

Parameter	Description	Value	Notes
D_A_	Nucleoid bound ParA-ATP diffusion constant	10^−2^ µm^2^/s	Fitted, can be increased without loss of qualitative behaviour of system. Nevertheless, it is difficult to physically reconcile more mobile nucleoid-bound ParA-ATP with the ability to immobilize a plasmid with a lower diffusion constant. Therefore we have assumed that ParA-ATP diffuses 10x slower than the plasmid, ensuring that the assumption that ParA-ATP can immobilize plasmids is physically justified.
D_P_	Plasmid diffusion constant	10^−1^ µm^2^/s	A relatively high value is needed for compatibility with previous experiments [9]. An upper bound on the plasmid diffusion constant from experiments (Fig. 3C) turned out to be too low for this model to fit our experimental observations.
k_on_	ParA-ATP nucleoid binding	50 s^−1^	Constrained by experiment [8].
k_AB_	ParA-ATP to plasmid binding	100 s^−1^	Fitted, should be high enough to allow for plasmid immobilization.
k_B_	Plasmid bound ParA-ATP hydrolysis (into ParA-ADP) stimulated by ParB.	68.5 s^−1^	Fitted together with D_A_ and k_W_ to ensure equal plasmid spacing.
k_W_	(Cytoplasmic) ParA-ADP to ParA-ATP conversion	1/15 s^−1^	Constrained by experiment [8], this value should be low enough to ensure that cytoplasmic ParA diffusion can generate a uniform cytoplasmic ParA-ATP and ParA-ADP concentration.

Prior work has demonstrated plasmid displacement along the long cell axis of up to 3–4 µm within 10 min [9], [15]. With a diffusion/immobilization mechanism all plasmid movement in between immobilization events is generated by (unbiased) free diffusion, for which we have (in 1d) a mean square displacement (MSD) of 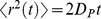. By inserting the above length and time scales into this equation, we conclude that a plasmid diffusivity of at least D_P_∼10^−2^ µm^2^s^−1^ would be required to generate sufficiently rapid diffusive movement in accordance with previous experiments. We therefore chose D_P_ = 10^−1^ µm^2^s^−1^. In order to physically justify that ParA can immobilise the plasmids, we chose the nucleoid bound ParA-ATP diffusivity to be lower than D_P_, with D_A_ = 10^−2^ µm^2^s^−1^ (Table 2). We experimentally constrained the overall copy number of ParA for pB171 *par2* by semi-quantitative Western blots, which revealed that there were approximately 8×10^3^ ParA monomers per cell (S1B Fig.). This diffusion/immobilization model could produce equal plasmid spacing on simulated growing nucleoids with varying numbers of plasmids (Fig. 2B,C, S3A). This result demonstrates that using a sufficiently high (low) plasmid (ParA) diffusivity, respectively, the equal plasmid spacing seen in our experiments (Fig. 1B,C,D, S2A,B Fig.) and previously [9], could in principle be achieved using a diffusion/immobilization mechanism.

### Free plasmid mobility is too low for a diffusion/immobilization mechanism

**Figure 3:**
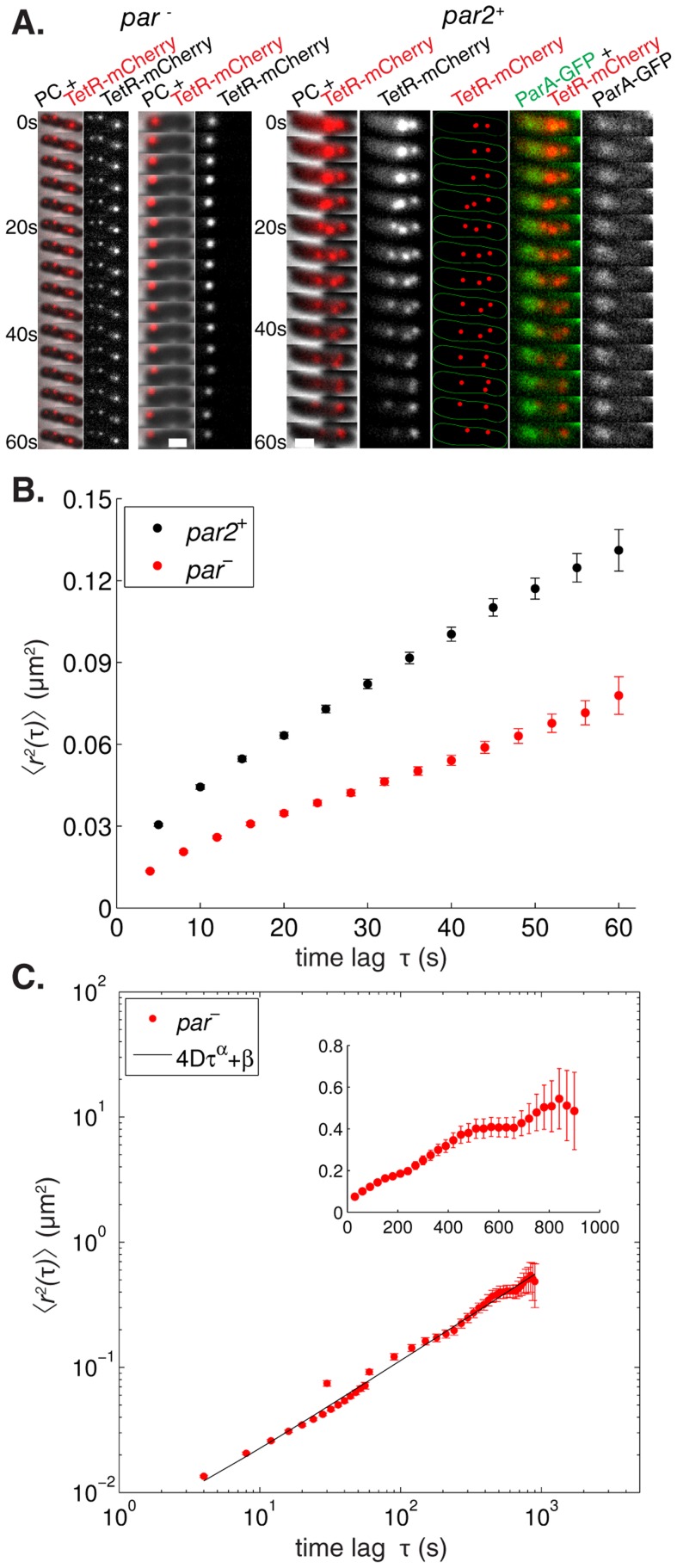
The *par2* segregation system increases plasmid mobility. (**A**) Time lapses showing the localization of *par^-^* pMH82tetO120 (mini-R1, *par^-^*, *tetO120*) and *par2^+^* pSR236 (mini-R1, *parC1^+^*, *parA^-^*, *parB^+^*, *parC2^+^*, *tetO120*, *P_lac_::parA::eGFP*) plasmids in *E. coli* cells harboring pSR124 (*P_BAD_::tetR::mCherry*). The *par2^+^* time lapse, with ParA-GFP localization, shows a segregation event where two foci segregate ≥0.8 µm further apart within 20 s. PC =  hase contrast, scale bar: 1 µm. (**B**) Mean square displacements 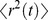 after time lag τ were extracted from plasmid trajectories (n_par-_ = 747, n_par2+_ = 763) using strains specified in (**A**), *par^-^* (red) and *par2^+^* (black), error bars: standard error of the mean. (**C**) Log-log plot of experimental mean square displacements 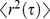 after time lag τ (red) were extracted from plasmid trajectories over 1 min as in (**A,B**) and (inset, linear scales, n = 50) over 15 min from *par^-^* pMH82tetO120 (mini-R1, *par^-^*, *tetO120*) plasmids in *E. coli* cells harboring pSR124 (*P_BAD_::tetR::mCherry*). At timescales on the order of 10 min saturation of the MSD occurs due to cellular confinement. A nonlinear least square fit (black line) using the function 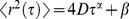 was used to estimate parameter values: α = 0.73±0.02, D = 9.7±1.3×10^−4^ µm^2^s^−α^, β = 1.6±2.4×10^−3^ µm^2^, (R^2^ = 0.99, p-values: 8×10^−15^, 8×10^−3^ and 0.50 respectively). See Materials and Methods for details; error bars: standard error of the mean.

To test whether the requirement of a relatively high free plasmid mobility is met *in vivo*, we compared the movement of test-plasmids with and without *par2*. We analyzed trajectories of labeled plasmid foci using the *tetO-*TetR-mCherry labeling system, measuring the positions over time (Fig. 3A) and MSDs for each time lag τ. Plasmid motion will be biased by a functional *par2^+^* partitioning system, in contrast to the random motion of *par^-^*. Nevertheless comparing MSDs can still be informative in comparing relative overall mobilities. On time scales up to a minute we found that the *par2^+^* MSD is higher than in *par^-^* (Fig. 3B), showing that, on average, *par2^+^* plasmids are more mobile than their *par^-^* counterparts. Note that the number of data points for the short time lags far exceeds the number of trajectories (n_par-_ = 747, n_par2+_ = 763), since every trajectory contains multiple short time lags. Consequently our estimates for the mean are relatively precise for short time lags. It is true that the error on the mean does not reflect inaccuracy due to experimental limitations in determining the actual plasmid position, for instance due to a finite pixel size. However, that error is the same for both *par2* ^+^ and *par* ^-^. Moreover, since the error is also time lag independent, it is taken into account in our fitting procedure as a time lag independent term (for more details see below and Materials and Methods). Overall, these results are hard to reconcile with a diffusion/immobilization mechanism where the *par2* system can only immobilize plasmids, and thus lower their MSD. These MSD values could in principle be limited due to cellular confinement. However, we found that MSD saturation only starts to occur at much larger length scales at times of up to 10 min (Fig. 3C). In the presence of *par2*, plasmids generally reside within the nucleoid region, while in its absence they tend to become somewhat more polar localized, although they can still sample the entire cell volume on long enough timescales [23]. Consistently we still find many *par^-^* plasmids located within the nucleoid region (S3B Fig.). Restricting the mobility analysis to *par^-^* plasmids within the nucleoid region did not alter the resulting MSD curves significantly (S3B Fig.). We conclude that the presence of *par2* can increase plasmid mobility in the nucleoid region, which is inconsistent with a diffusion/immobilization mechanism. We emphasize that this conclusion can be made irrespective of the underlying (*par^-^*) plasmid transport processes, which we now describe in more detail.

It has been reported that chromosomal loci and RNA-protein particles exhibit subdiffusive, rather than diffusive, behavior in the cytoplasm [24], [25]. Therefore it is possible that plasmids without a segregation mechanism could also exhibit subdiffusive motion. Further analysis is required to fully distinguish subdiffusion from the additional effects of cellular confinement or glass-like properties of the bacterial cytoplasm [23], [25]. Nevertheless such additional analysis is not required for the conclusions on *par^-^* plasmid mobility relevant to this study, as we now explain. Subdiffusion results in an expected MSD displacement of the form 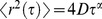, with α<1 and D the apparent diffusion constant (in units of µm^2^s^-α^). We find that our MSD displacements on both short and long timescales are well described by subdiffusion with α = 0.7–0.8 and an apparent diffusion constant D = 5–10×10^−4^ µm^2^s^−α^ (Fig. 3C and Materials and Methods for details). This is consistent with other recent reports on *par^-^* plasmid mobility [23], [26]. Importantly the experimental MSD is lower on all observed timescales than a hypothetical particle that would perform free diffusion inside a cell with a diffusion constant D_f_ = 10×10^−4^ µm^2^s^−1^. This upper limit is already much lower than that needed to be consistent with the previously reported plasmid displacement data discussed above. We will further exploit this upper limit in our analysis below.

To further investigate the effect of *par2* on plasmid positioning, we also studied rapid plasmid segregation events. We defined these as cases where two plasmid foci whose separation is initially ≤0.3 µm, move within 20 s at least another 0.8 µm apart (Fig. 3A, S3C). We also allowed for the two foci to be initially merged. Using these criteria, despite equally large data sets, we found 13 such events in *par2^+^* and only one such case in *par^-^*. Furthermore, we only retrieved 2 further *par2^+^* segregation events when we relaxed the criterion to separation within 60 s instead of 20 s. This analysis shows that most segregation events occur rapidly. When we investigated the 26 plasmid trajectories involved they showed larger maximal MSDs compared to sets of 26 trajectories that were repeatedly randomly sampled from the whole *par2^+^* dataset (p<10^−6^). This finding indicates that the *par2* system can particularly enhance the mobility of plasmids when they are in close proximity. We then simulated 300 plasmid duplication events with our diffusion/immobilization model to determine the magnitude of diffusion constant required to generate the experimentally observed segregation. Note that we used diffusion rather than subdiffusion here because we have already determined that *par^-^* plasmid movement is slower on all observed timescales than free diffusion with a diffusion constant D_f_ = 10×10^−4^ µm^2^s^−1^. Hence, if the required diffusion constant is larger than D_f_ then we have also ruled out a subdiffusion/immobilization model. We required that 5% (15 out of 300) of segregated distances within 20 s were at least 0.8 µm (a very conservative requirement, since the criterion was satisfied by 13 of our 15 experimental segregation events). This requirement necessitated a free plasmid diffusion constant on the order of 10^−1^ µm^2^s^−1^, about two orders of magnitude higher than our experimentally observed upper bound D_f_ on the experimental *par^-^* plasmid mobility. Hence, we conclude that the plasmids are generally too immobile for a diffusion/immobilization (or subdiffusion/immobilization) mechanism to explain these segregation events. Also the qualitative behaviour of segregation events in the diffusion/immobilization model appears different, since experimental segregation events (Fig. 3A, S3C) show more directionally biased motion, while the diffusion/immobilization model generates more sustained random, diffusive motion during segregation, prior to immobilization at equally spaced positions (Fig. 2B). Nevertheless, these segregation events were sufficiently rare not to significantly alter the overall MSD behaviour of the entire dataset shown in Fig. 3B. Thus the increased average mobility in the presence of *par2^+^* cannot only be ascribed to these segregation events.

It is possible that the *tetO-*TetR-mCherry labeling system caused reduced plasmid mobility as compared to unlabelled plasmids. However, as we used the same labeling method for both *par2^+^* and *par^-^* cases, our above conclusions on relative mobility are unaffected. Moreover, our *tetO-*TetR-mCherry labeled plasmids still exhibited rapid segregation events (such as in Fig. 3A), underscoring the ability of *par2* to overcome low plasmid mobility. Overall, we find that diffusion/immobilization cannot explain our data on *par2* ^+^ versus *par^-^* plasmid mobility, as well as on rapid *par2* ^+^ plasmid segregation.

### ParA structures competing to direct plasmid motion can space plasmids equally over the nucleoid

Given the shortcomings of the diffusion/immobilization model, we next tested models based on directed motion, allowing more rapid directed rather than unbiased diffusive plasmid movement. More specifically, we tested models based on the formation of competing ParA polymers, with ParB-*parC*-stimulated ParA-ATP hydrolysis directing plasmid movement. By modulating the length of these polymers, we thereby tested the robustness of directed motion models to generate equal plasmid positioning.

**Figure 4:**
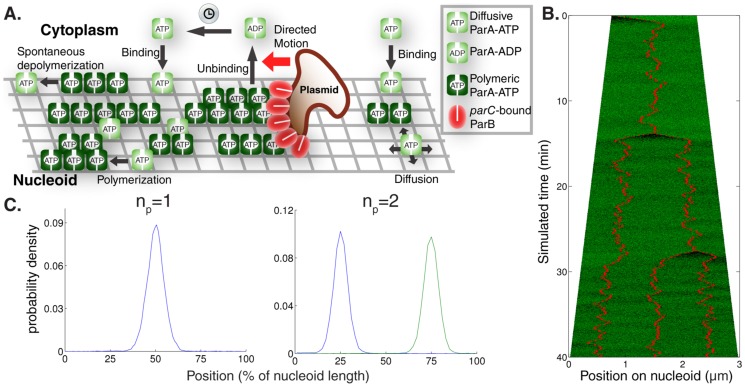
The directed motion model can move and maintain plasmids at equally spaced positions. (**A**) Schematic illustration of *par2* directed motion model. The clock indicates the slow conversion of cytoplasmic ParA-ADP into cytoplasmic ParA-ATP that is competent to bind to the nucleoid. (**B**) Typical simulation kymograph of directed motion model with short polymers for a simulated growing cell where a plasmid (red) is initially directed from a nucleoid edge to mid-cell by ParA (green) filament competition. After plasmid duplication, the system dynamically self-organizes to attain equal spacing. (**C**) Time-averaged plasmid position distributions for directed motion model with short polymers for n_p_ = 1,2 plasmids on a simulated nucleoid growing from 1.5 µm to 3 µm in 40 min without plasmid duplication. Plasmid distributions were obtained by sampling positions every 5 s in 36 independent simulations.

We again used a Gillespie algorithm to simulate ParA dynamics on the nucleoid (see Fig. 4A, Materials and Methods and Tables 3,4 for details). The nucleoid was represented as a rectangular lattice (dx = 5 nm in both dimensions), with a much shorter width (30 nm) than length (several µm). Similar reactions as in the diffusion/immobilization model described the cytoplasmic dynamics of ParA-ADP and ParA-ATP. Nucleoid-associated ParA-ATP could also still diffuse across the nucleoid in a mobile state in all four directions to neighbouring sites with diffusion constant *D*_A_. However, two of these molecules at sites neighboring each other along the long nucleoid axis could interact to form a ParA polymer of two subunits, with reaction parameter k_p_. Further ParA-ATP polymerization could occur by attachment of mobile ParA-ATP, located at a site immediately next to the tip of an existing ParA polymer, but only along the long axis. ParA-ATP polymers were assumed to be immobile. A ParA-ATP polymeric subunit could depolymerize spontaneously with reaction parameter k_dp_, i.e. be converted into a mobile ParA-ATP at the same site. Given that its size is similar to the width of the lattice, we only took into account the plasmid position along the long axis and we assumed that it occupied all sites along the short axis simultaneously. The plasmid could diffuse with our experimentally estimated diffusion coefficient *D*_P_ along the long axis when polymeric ParA-ATP was not present either at any of the sites that the plasmid occupied or sites neighbouring the plasmid. In the presence of polymeric ParA-ATP, the plasmid was assumed to be tethered to such a polymer (via a ParB-*parC* complex), which prevented plasmid diffusion. At sites with a plasmid present, polymeric ParA-ATP could be converted into cytoplasmic ParA-ADP with reaction parameter k_B_. Reflecting directed motion, at sites neighbouring a plasmid occupied by polymeric ParA-ATP, a plasmid could with reaction parameter k_dm_ move to the coordinate along the long axis of that ParA-ATP subunit, coinciding with conversion of that ParA-ATP into cytoplasmic ParA-ADP. For wild-type simulations, any plasmid in the system formed a hard wall to mobile ParA-ATP diffusion so that diffusing ParA-ATP molecules could not diffuse past a plasmid.

**Table 3:** Reactions and propensities used in the directed motion models.

Reactions	Propensities p_t_
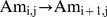 , 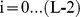 , 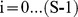	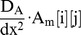 , if 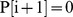 0, otherwise
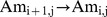 , 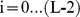 , 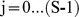	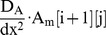 , if 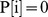 0, otherwise
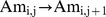 , 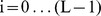 , 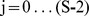	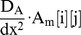
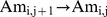 , 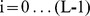 , 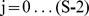	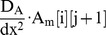
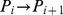 , 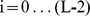	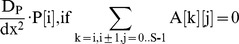 0, otherwise
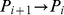 , 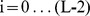	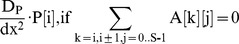 0, otherwise
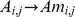 , 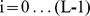 , 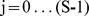	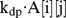 , if 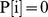 0, otherwise
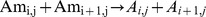 , 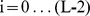 , 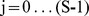	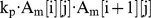 , if 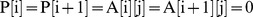 0, otherwise
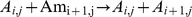 , 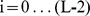 , 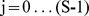	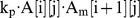 , if 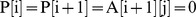 0, otherwise
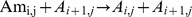 , 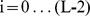 , 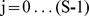	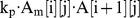 , if 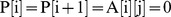 0, otherwise
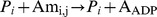 , 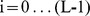 , 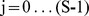	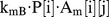
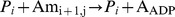 , 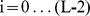 , 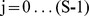	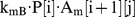
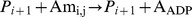 , 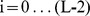 , 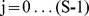	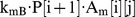
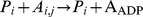 , 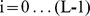 , 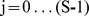	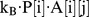
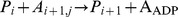 , 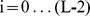 , 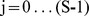	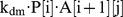
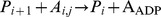 , 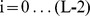 , 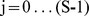	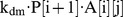
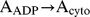	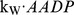
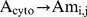 , 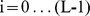 , 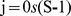	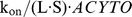

**Table 4:** Parameter values used in the directed motion models.

Parameter	Description	Value	Notes
D_A_	Nucleoid bound ParA-ATP diffusion constant	1 µm^2^/s	Constrained by experiment [18], value can be varied by several orders of magnitude without loss of qualitative behaviour of system. Note that this form of ParA-ATP does not have an effect on the mobility of plasmids, since only polymeric ParA-ATP, immobile due to the interaction with the nucleoid, can direct the motion of a plasmid.
D_P_	Plasmid diffusion constant	3×10^−4^ µm^2^/s	Constrained by experiment (Fig. 3C).
k_on_	ParA-ATP nucleoid binding	50 s^−1^	Constrained by experiment [8].
k_B_	Plasmid bound polymeric ParA-ATP hydrolysis (into ParA-ADP) stimulated by ParB.	68.5 s^−1^	Chosen to be the same as k_B_ in diffusion/immobilization model for consistency; constrained by k_B_ ≫ k_dm_ which ensures that all the ParA-ATP at the location of a plasmid is converted into cytoplasmic ParA-ADP before the plasmid moves to a neighboring site. Value can be varied within a wide range without loss of qualitative behaviour of system.
k_mB_	Plasmid bound mobile ParA-ATP hydrolysis (into ParA-ADP) stimulated by ParB.	40 s^−1^	Fitted, value can be varied within a wide range without loss of qualitative behaviour of system. Setting this rate too high depletes ParA-ATP locally around a plasmid, which inhibits directed plasmid motion events.
k_W_	(Cytoplasmic) ParA-ADP to ParA-ATP conversion	1/15 s^−1^	Constrained by experiment [8], this value should be low enough to ensure that cytoplasmic ParA diffusion can generate a uniform cytoplasmic ParA-ATP and ParA-ADP concentration.
k_dm_	Plasmid directed motion rate (in presence of one neighboring plasmid)	0.8 s^−1^	Constrained by experiment (Fig. 2A). If interpreted as biased plasmid diffusion along the polymer (burnt-bridge mechanism [4]), this would result effectively in a maximal plasmid diffusion constant of 1×10^−4^ µm^2^s^−1^(short) and 1.2×10^−4^ µm^2^s^−1^ (long). These values are consistent with the free diffusion constant D_P_ (see above), since the interaction with immobile ParA-ATP polymers could lower the plasmid mobility.
k_p_	Polymerization: mobile ParA-ATP to polymeric ParA-ATP conversion	800 s^−1^ (short), 10^6^ s^−1^(long)	Fitted together with k_dm_ and k_W_ to ensure equal plasmid spacing. k_p_ and k_dp_ together with the total ParA-ATP concentration determine the extent of ParA-ATP polymerization.
k_dp_	Spontaneous depolymerization: ParA-ATP to mobile ParA-ATP conversion	10 s^−1^(short), 10^−4^ s^−1^(long)	Fitted. See notes on k_p_ parameter above.
S	Short axis length of the nucleoid region where nucleoid bound ParA-ATP can polymerize.	30 nm (short), 25 nm (long)	Fitted, values should be small compared to the long nucleoid axis length to ensure that segregation occurs along the long nucleoid axis.
	**Perturbed nucleoid simulations**		Parameter values as above unless specified below. See also Materials and Methods for further details.
k_mB_	Plasmid-bound mobile ParA-ATP hydrolysis (into ParA-ADP) stimulated by ParB.	4 s^−1^	Fitted, value is chosen to simulate the effect of a disordered nucleoid structure, allowing mobile ParA-ATP to diffuse past plasmids.
S	Short axis length of the nucleoid region where nucleoid bound ParA-ATP can polymerize.	30 nm (short), 10 nm (long)	Fitted, values are chosen to ensure a sufficient amount of mobile ParA-ATP.

We first adjusted the ParA-ATP polymerization rate to generate short filaments, of approximately 10 subunits in length (Table 4 for parameters). Simulations again faithfully reproduced the equal spacing of plasmids along simulated growing nucleoids with varying numbers n_p_ of plasmids (Fig. 4B, n_p_ = 1,2 in Fig. 4C, n_p_ = 3,4 in S4A Fig.) in good agreement with our experiments (Fig. 1, S2). By adjusting the ParA-ATP polymerization rate (Table 4), long continuous ParA polymer bundles could also be generated. In that case equal spacing could also be achieved (S4B,C Fig.). Intuitively, in both short and long filament cases, this occurs because in an irregularly spaced plasmid configuration, the unequal ParA concentrations on either side of a plasmid result in an unequal degree of ParA polymerization. This in turn results in an unequal amount of competitive directed motion events to each side, resulting in effective directed translocation over longer length scales back towards an equally positioned state. Plasmid separation occurs when two nearby plasmids encounter two ParA-ATP structures extending in opposite directions away from the plasmids. The two ParA-ATP structures will then necessarily mediate a segregation event. The effect of directed movement in this model is clearest in the case of plasmid segregation events (Fig. 4B, S4B), where we see rapid segregation consistent with the fast segregation events observed experimentally (see Fig. 3A).

### ParA-GFP oscillations are not continuously required for equal plasmid spacing

Intriguingly, simulations of the directed motion model did not generally produce sustained spatiotemporal oscillations of ParA across the nucleoid (short polymers: Fig. 4B, long polymers: S4B Fig.). A lack of sustained oscillations would therefore appear to be a common feature of models where competitive ParA structures generate equal plasmid spacing. This absence was unexpected, as prior experimental work had emphasized the oscillatory aspect of the ParA dynamics [12]–[14]. To experimentally test this key model prediction in an unbiased fashion, we experimentally measured the degree of ParA asymmetry in the *par2* system in a large dataset (n = 134) of snapshots of ParA-GFP across the nucleoid. We examined only cases with a single plasmid *tetO-*TetR-mCherry focus, where sustained oscillations should be easiest to infer. The ParA-GFP fluorescence signal from pole to plasmid position was summed and divided by the respective pole-to-plasmid distance. This generated two ParA-GFP fluorescence densities I_L_ and I_R_ for either side extending to the two cell poles. This allowed us to compute the normalized asymmetry measure |I_L_-I_R_|/|I_L_+I_R_| [27] for ParA (see Materials and Methods for details). Asymmetric ParA-GFP distributions, arising for example from oscillations, where for example I_L_≈0, I_R_≈1, will give asymmetry values closer to one, whereas symmetric ParA-GFP distributions, where I_L_≈I_R_, will give values closer to zero. Note that the ParA-GFP exposure time used here was 1.5 s; clearly, we cannot measure asymmetries that occur on a timescale faster than this exposure time. However, the timescales of the plasmid and ParA-GFP dynamics are on the order of tens of seconds or longer and it is therefore unlikely that any significant asymmetry is being missed by our measurements.

**Figure 5:**
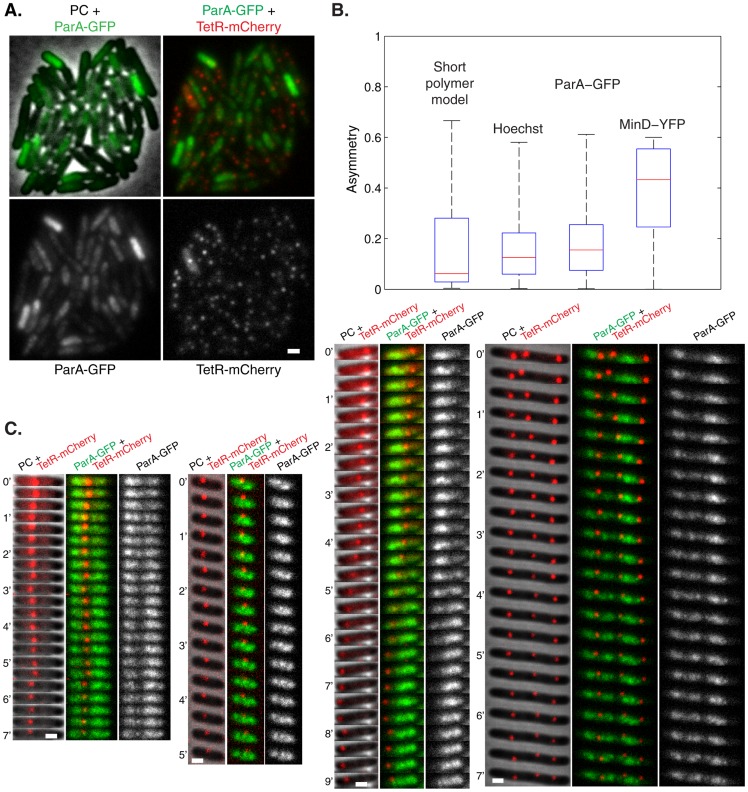
As predicted by the directed motion model, ParA-GFP distribution is relatively symmetric. (**A**) Localization of plasmids and summed Z-stack of ParA-GFP distributions in a field of cells. Scale bar: 1 µm; plasmid: pSR233 (mini-R1, *par2^+^*, *P_lac_::parA::eGFP*, *tetO120*) in *E. coli* cells harboring pSR124 (*P_BAD_::tetR::mCherry*). (**B**) ParA-GFP (n = 134) asymmetry measure |I_L_-I_R_ |/|I_L_+I_R_| using fluorescence densities I_L_, I_R_ on left, right sides of a plasmid focus along long cell axis (see Materials and Methods). Comparison shown to the prediction of directed motion model with short polymers, Hoechst (n = 134) and MinD-YFP case [27]. Box plots represent minimal, first quartile, median, third quartile and maximal values of asymmetries in all cases. (**C**) Time lapses showing localization of *par2^+^* pSR236 (mini-R1, *parC1^+^*, *parA^-^*, *parB^+^*, *parC2^+^, P_lac_::parA::eGFP, tetO120*) plasmids in *E. coli* cells harboring pSR124 (*P_BAD_::tetR::mCherry*).

When we examined our whole distribution of cells exhibiting single plasmid *tetO-*TetR-mCherry foci, we found that the degree of ParA-GFP asymmetry (Fig. 5A,B) was low in comparison with the well-established MinD spatiotemporal oscillator [27]. Furthermore, the ParA-GFP asymmetry did not correlate with cell length (S5A Fig., R^2^ = 0.08), unlike the case of MinD-YFP [27]. We also compared the ParA-GFP asymmetry to the Hoechst signal. This DNA stain labels the nucleoid itself, which is relatively uniform along the long cell axis [28]–[30]. Here, any asymmetry is not expected to depend on the plasmid foci positions. The Hoechst asymmetry distribution was indeed concentrated around relatively small values, but was apparently measurable within our approach (Fig. 5B, S5B). Importantly, we found that the ParA-GFP asymmetry measure had a similarly low value as for the Hoechst case (Fig. 5B, S5B, no significant difference, Kolmogorov-Smirnov test), and that for both the asymmetry is uncorrelated to the plasmid focus position (S5C Fig.). We therefore conclude that for a single plasmid focus, ParA-GFP typically resides on both sides of a plasmid, with relatively little asymmetry or oscillation, as predicted by the directed motion model, irrespective of a weak (Fig. 5B) or strong (S5B Fig.) degree of polymerization.

Previous analyses had focused on plasmids migrating in the wake of retracting ParA-GFP structures [9]. Such events can transiently give rise to relatively high ParA-GFP asymmetries (see, for example, Fig. 3A, 5C). Accordingly, we conclude that ParA asymmetry or oscillations are not continuously required for *par2* mediated plasmid positioning. Transient asymmetry, including oscillations, instead likely arises from the dynamics needed to bring about equal plasmid spacing following a spatial perturbation or plasmid duplication event (Fig. 5C). Once the ParA distribution has returned to being relatively symmetric, this coincides with an equally spaced plasmid configuration (Fig. 5C). Such dynamics can be seen in our model simulations (Figs. 4B, S4B): asymmetric during plasmid segregation events, but relatively symmetric otherwise. This analysis can therefore accommodate both our findings of a relatively symmetric ParA distribution with previous reports emphasizing asymmetry and oscillations. Overall, our finding of predominantly symmetric, non-oscillatory ParA dynamics may help to reconcile similar findings for ParA in other plasmid partitioning systems, such as for plasmid P1 [15], [16].

### ParA-GFP forms structures within the nucleoid region

**Figure 6:**
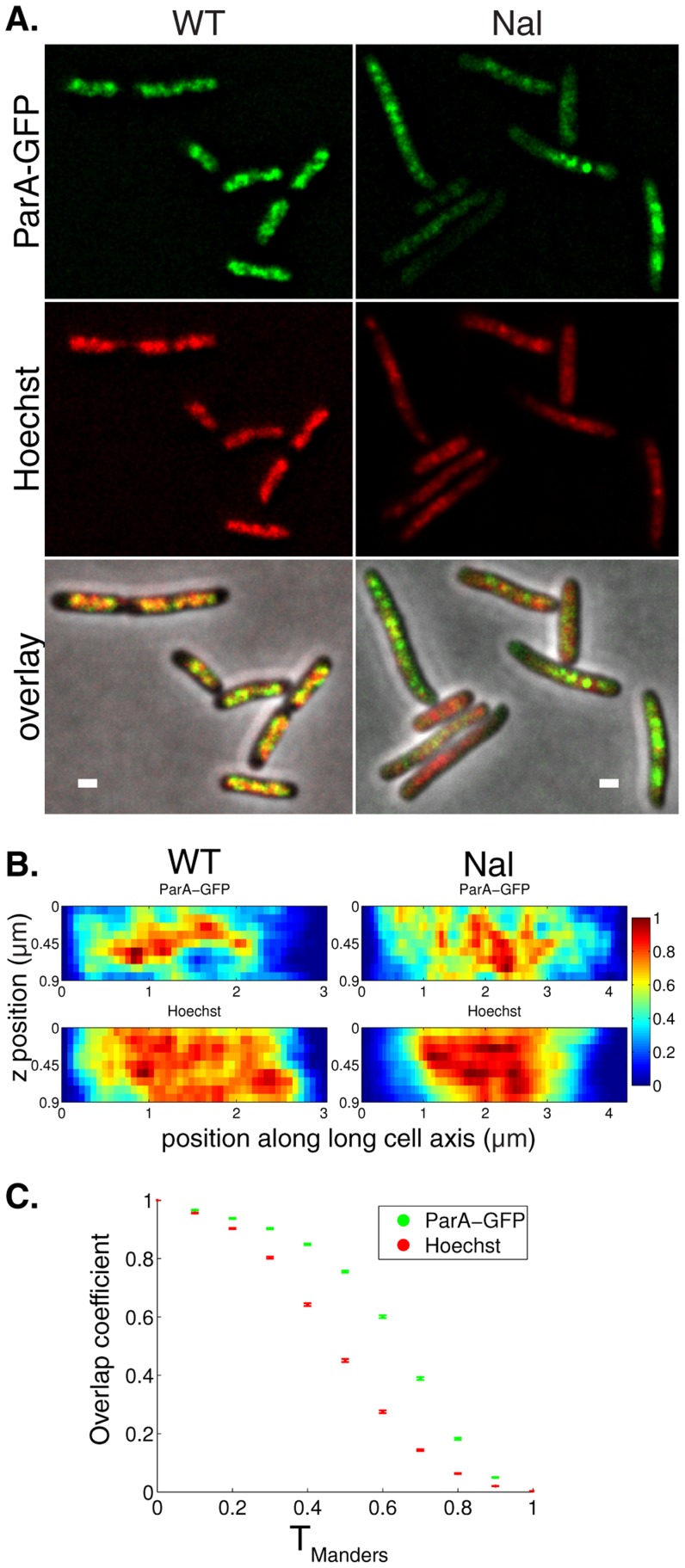
ParA forms structures within the nucleoid region. (**A**) Fluorescence localization of ParA-GFP (green), Hoechst DNA stain (red) and overlay, at mid-height through cell, taken from deconvolved Z-stacks showing structures that are disrupted with 50 µg/ml nalidixic acid treatment (Nal) compared to WT. Scale bar: 1 µm; plasmid: pGE230 (mini-R1, *par^-^*, *P_lac_::parA::eGFP*). (**B**) Normalized fluorescence intensity profiles along the long cell axis for 9 in focus z heights (dz = 0.1 µm) resulting from deconvolved Z-stacks in representative WT and Nal-treated strains. (**C**) Manders overlap coefficients in WT cells (error bars: standard error of the mean, n = 678) showing the fraction of ParA-GFP fluorescence intensity that overlaps with Hoechst DNA stain when the latter is above a threshold T_Manders_ (ParA-GFP, green) and the reverse (Hoechst, red). ParA-GFP overlaps more with Hoechst DNA stain (p-values ranging from 10^−12^ to 10^−132^, see Materials and Methods) than the reverse.

One required feature to achieve equal plasmid spacing is that the ParA-ATP should be organized in a 1d-like structure along the nucleoid as concluded above. However, it is unclear why ParA-ATP on either side of a plasmid would align in a coherent 1d-like structure with their ends coinciding with a plasmid. One potential explanation for this 1d-like behavior is that the ParA-ATP structures are sensitive to the overall nucleoid architecture. To test these features, we examined the localization of ParA-GFP and Hoechst signal simultaneously using optical sectioning in WT cells (n = 678) without *par2-*carrying plasmids to prevent dynamic ParA-GFP structure disassembly. ParA-GFP intensity correlated well with the DNA stain (Fig. 6A,B, S6A, Pearson's correlation coefficient r_P_ = 0.81), indicating that ParA-GFP localization was indeed dependent on the underlying nucleoid. Importantly, ParA-GFP overlaid more with Hoechst than the reverse (Fig. 6C), indicating that ParA forms structures within the nucleoid region rather than uniformly covering the nucleoid. Although the resolution of our techniques does not allow identification of potential individual ParA polymers, in many cases we did observe extended 1d-like ParA-GFP structures on the nucleoid (Fig. 6B, S6A). Care must be taken in interpreting fluorescent localization studies due to potential artifacts, for example GFP-induced polymerization [31]. However, wild-type plasmid loss rates and plasmid foci positioning in cells expressing ParA-GFP argue against localization or polymerization artifacts in our case [9], [12].

### Equal plasmid spacing is compromised in cells with a perturbed nucleoid

**Figure 7:**
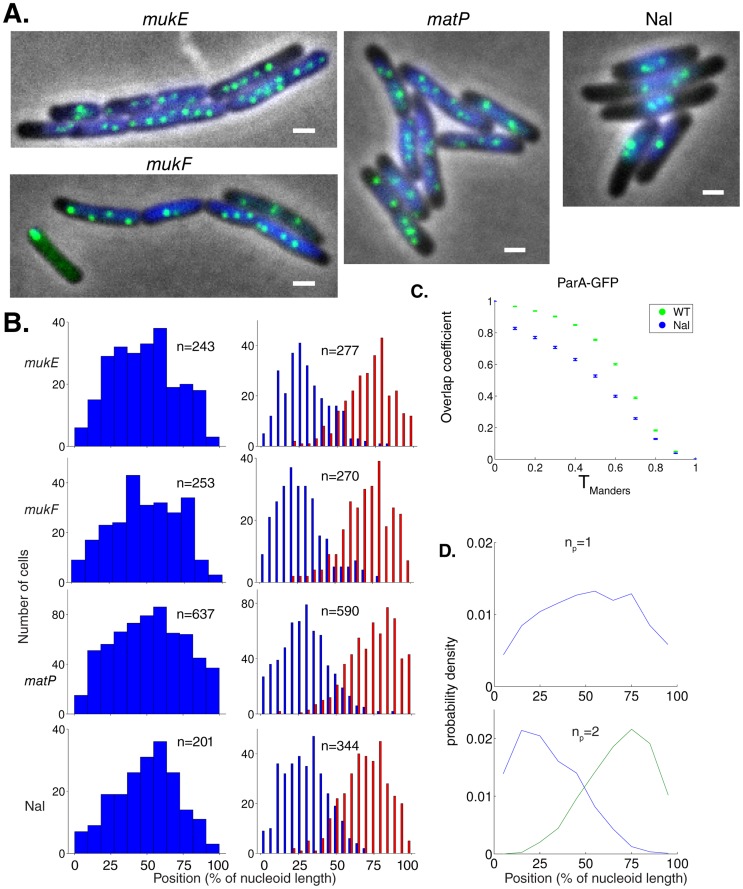
Nucleoid morphology disruption causes aberrant plasmid focus positioning. (**A**) Fluorescence localization of ParB-GFP (green) and Hoechst DNA stain (blue) in *mukE*, *mukF*, *matP* mutants and wild-type cells treated with 50 µg/ml nalidixic acid (Nal). Scale bar: 1 µm; plasmid: pFS21 (mini-R1, *parC1^+^*, *parA^+^*, *parB::sfGFP*, *parC2^+^*). (**B**) Histograms of plasmid foci positions (n_p_ = 1,2) for mutants/treatments described in (**A**) relative to nucleoid size. According to Kolmogorov-Smirnov tests, all distributions are broader than WT (Fig. 1C) with p<10^−2^ except Nal n_p_ = 1: p<0.05. (**C**) Manders overlap coefficients (error bars: standard error of the mean) of ParA-GFP comparing WT (n = 678) and Nal-treated cells (n = 862). Consistent with a decrease in the Pearson's correlation coefficient r_P_ (p<10^−38^), ParA-GFP overlaps less with Hoechst in Nal-treated cells as compared to WT (p-values ranging from 10^−51^ to 10^−144^). (**D**) Time-averaged plasmid position distributions for directed motion model with short polymers obtained as in Fig. 2C from 124 independent simulations. Here, mobile DNA-bound ParA-ATP was now able to diffuse past a plasmid.

We reasoned that if ParA structures are reliant on the nucleoid morphology for their organization, then mutants/treatments that perturb the overall nucleoid structure should also exhibit alterations in ParA localization and therefore plasmid focus positioning (Fig. 7A). We measured plasmid focus positioning in *mukE*, *mukF* and *matP* mutant strains, as well as in cells treated with the DNA gyrase inhibitor nalidixic acid (Nal), all of which exhibit defects in nucleoid organization [32]–[34]. Nucleoid length distributions were altered in all of these cases (S7A Fig.) and, consistent with our hypothesis, there was in each case a similar deterioration in the fidelity of plasmid focus positioning (n_p_ = 1,2 in Fig. 7B, S7B,C, n_p_ = 3,4 in S7B,C,D Fig.) towards a random distribution (S7E Fig.). This deterioration may not have been large enough to detect in stability assays [35], [36]. Similarly, in *E. coli mukB* mutants, perturbed plasmid positioning without compromising plasmid stability has also been observed, although for the segregation mechanism mediated by ParM [37]. The deteriorations in plasmid positioning could have resulted from other effects, such as an induction of the SOS response in Nal-treated cells. However, the similarity of the altered plasmid positioning in all four cases instead suggests a common positioning defect based on nucleoid perturbation. This deterioration could also be due to an altered plasmid structure. However, at least for the case of *matP* we do not favor this hypothesis, due to the absence of MatP target sites (*matS*) on our test-plasmid.

To provide evidence that the above deterioration in plasmid positioning arose from an altered ParA distribution, we systematically examined localization of ParA-GFP and Hoechst stain simultaneously in Nal-treated cells (n = 862), which had the largest visible perturbations. We were able to quantify (Fig. 7C, S6B, p<10^−149^, see Materials and Methods) perturbations in nucleoid structure that were detectable by eye (Fig. 6A, S6A). Moreover, visual inspection showed that the ParA-GFP distribution followed the nucleoid structure less closely than in the WT (Fig 6A, S6A). This finding was quantitatively confirmed by a correlation coefficient of r_P_ = 0.68, decreased from its WT value of 0.81 (p<10^−34^), and also by a decrease in the ParA-GFP overlap coefficient (Fig. 7C). Altogether, these findings support our hypothesis that the nucleoid provides a template for 1d-like ParA-ATP structure formation, which is partially compromised when the nucleoid structure is perturbed.

To reproduce this behavior in the directed motion model, we assumed that mobile DNA-bound ParA-ATP could now diffuse past a plasmid (see Materials and Methods for details). This could be due to the disordered nucleoid structure resulting in a deteriorated ParA-ATP structure organization, thereby allowing ParA-ATP to spatially bypass ParB-*parC* complexes and compromise the ParA concentration differences between either side of a plasmid. The directed motion model with a weak (Fig. 7D, S7F Fig.) or strong (S7F Fig.) degree of polymerization could then reproduce the observed plasmid focus distributions (Fig. 7B).

## Discussion

Stable DNA inheritance is important for the viability of essentially all organisms. In bacteria, the *parABC* genes have a major role in this process for plasmid DNA [1]. In this study, we have investigated how *E. coli* utilizes the *par2* partitioning system from plasmid pB171. We have for the first time provided a robust mechanistic explanation for how plasmids are equally spaced over the nucleoid, a process vital for the fidelity of low copy number plasmid inheritance. We propose that competing ParA structures function to direct plasmid movement over the nucleoid to equally spaced positions. This mechanism is likely relevant to other *parABC* systems that move and position sub-cellular objects.

It has previously been proposed that plasmid positioning is controlled by concentration gradients of ParA-ATP over the nucleoid, caused by plasmid-associated ParB-*parC* complexes mediating ParA-ATP hydrolysis [8], [16]–[20]. In this so-called diffusion-ratchet mechanism [8], [17], [18], [20], it has remained unclear whether such a mechanism could actually mediate equal plasmid spacing, and if so, which specific properties of the system were key. In particular, it was left unclear how ParA actually influenced plasmid movement [8], [17], [18], e.g. through immobilizing plasmids or actively directing their motion through a chemophoresis force [19], [20]. Furthermore, although the diffusion-ratchet mechanism did not strictly preclude some degree of ParA polymerization, its gradient-aspect was emphasized as opposed to polymerization [8], [16]–[18], [20], leaving open the potential importance of polymerization. To provide elucidation of these key issues, we have therefore performed a mathematical analysis, which has led to predictions that we have experimentally verified.

We found that ParA-ATP nucleoid-binding, followed by diffusion over the nucleoid, and subsequent ParB-*parC*-stimulated ParA unbinding in a 1d model, is sufficient to generate dynamic ParA-ATP concentration gradients on either side of a plasmid. We have further shown that these ParA concentrations on either side of a plasmid are only symmetric in the case of equally spaced plasmids; unequally-spaced plasmid configurations will cause the ParA gradient to be steeper on one side rather than the other. Fundamentally, this asymmetry arises from two key properties: (i) a greater space for binding of ParA on one side as opposed to the other in unequally-spaced configurations, and (ii) ParA only being returned to the cytoplasm at discrete plasmid positions occupied by ParB-*parC*. The combination of these two features leads to the ParA density being increased in larger versus smaller inter-plasmid regions and hence to asymmetric ParA concentrations in unequally spaced plasmid configurations. According to our analysis, all that is then required for equal plasmid spacing is that the plasmids have a means to preferentially move up the locally steepest ParA concentration gradient and thus locate the equally spaced configuration with symmetric, competitive ParA concentrations around each plasmid. The exact means of plasmid translocation is therefore not critical; all that is important is that such movement can occur.

With this general framework established, we then investigated which specific means of plasmid movement up a concentration gradient were possible, and which was implemented for the *par2* segregation system. We first developed a diffusion/immobilization model and found that such a model could indeed lead to plasmid movement up a ParA gradient, as the plasmid tends to become trapped in regions of higher ParA concentration. However, when we tested this model experimentally, its predictions did not verify: in particular, plasmid mobility was higher in the presence rather than the absence of *par2*, and overall free plasmid mobility was too low to allow the experimentally-observed rapid plasmid segregation following duplication events. This intrinsically low mobility agrees with earlier measurements [23], [26], [38] and is likely a general feature for relatively large intracellular components, given the glass-like properties of the cytoplasm [23].

We then considered active means of ParA-mediated plasmid movement. In particular, we assumed that ParA-ATP could form polymeric filaments, which could subsequently depolymerize through the action of plasmid-associated ParB-*parC*. In this case, ParA-ATP could bind to the nucleoid, diffuse and then subsequently polymerize to form gradients of ParA polymers, with the degree of polymerization influenced by the overall ParA concentration at a particular location. We found that ParA polymer models could naturally explain enhanced plasmid mobility in the presence of *par2*, as well as rapid plasmid segregation events, much more satisfactorily than the diffusion-immobilization model, regardless of whether long or short ParA polymers were formed. This finding in particular shows that our directed motion model is sufficiently general to explain equal plasmid spacing as found in various *parABC* systems with different extents of ParA polymerization [8], [9], [18]. In addition, we note that this mechanism does not critically depend on ParA-ATP binding to the nucleoid as a dimer. A scenario where ParA polymerizes to a certain extent cytoplasmically, and subsequently binds and diffuses on the nucleoid before polymerizing further into immobile filaments, could also suffice.

A key aspect of our models is competition between ParA structures on either side of a plasmid to direct plasmid movement. Therefore our model predicts a comparatively symmetric ParA distribution on average, a prediction which we experimentally verified. We note here that such competition makes the system dynamics robust to alterations in ParA expression levels, since it is only the relative rather than absolute ParA levels on either side of a plasmid that are critical. This analysis potentially explains why cells with variable amounts of ParA-GFP (S1C Fig.), still possess functional segregation systems with low plasmid loss rates [9].

In the above polymer models, the movement of a plasmid is assumed to be directed by retracting ParA structures. The precise nature of this short-ranged directed motion is not specified by our analysis, and could include locally biased plasmid diffusion along a retracting polymer in a “burnt-bridge” mechanism [4] or even direct pulling [39]. This arbitrariness is a special case of our more general result that the mechanism by which a plasmid is able to move up a ParA concentration gradient is not important, only that such movement is possible. Other mechanisms of directed motion are also plausible. One possibility is that ParA-ATP does not polymerize at all, but nevertheless forms dense structures on the nucleoid with many ParA-ATP contacting a plasmid at any given time. In this variant, biased diffusion through an analog of a “burnt-bridge” mechanism is still possible. Another possibility is a DNA-relay, where directed motion is generated by the elastic dynamics of the nucleoid DNA to which ParA-ATP dimers are bound [21]. Moreover, plasmid diffusion seems not always required for directed plasmid movement. Brownian dynamics simulations based on ParB-*parC*-mediated disassembling ParA polymer bundles can both tether and pull plasmids simultaneously without the need for plasmid diffusion [39]. We propose that distinct underlying translocation mechanisms, as exemplified above, could be responsible for directed motion in different *parABC* systems and yet still attain similar equal plasmid spacing.

For our models to generate equal plasmid spacing, ParA should be organized into a 1d-like configuration along the nucleoid. If ParA were not organized in this way, it would be possible for ParA to diffuse around the sides of a plasmid without encountering the hydrolyzing effect of the ParB-*parC* complex. This would equalize the ParA concentrations on both sides even in the case of asymmetrically placed plasmids, leading to failure of the equal spacing mechanism. Potentially such ParA structures could consist of long ParA polymer bundles, or an extended region containing short ParA polymers or dimers. Importantly, in this work, we have provided experimental evidence for such ParA structure formation within the nucleoid region. Interestingly, it has been reported that the *E. coli* chromosome adopts a helical shape [28], [30]. Potentially the ParA structures could be preferentially located within a "valley" in this configuration, thereby naturally generating a 1d-like appearance, even for dimers or short polymers. Consistent with these concepts, we found experimentally that plasmid positioning is compromised in nucleoid perturbed strains. ParA structures could also provide a high enough ParA concentration to ensure plasmid tethering and directed plasmid motion, whilst preventing plasmids from diffusing away from the nucleoid, a process which would compromise regular positioning. Further investigation of the exact involvement of the nucleoid in intracellular cargo positioning is therefore an important future goal.

## Materials and Methods

### Diffusion/immobilization model

On the one dimensional lattice with sites of size dx = 5 nm, sites are numbered 0… (L-1). Reactants are 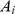: ParA-ATP at site i with number 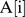 (≥0), 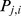: plasmids with j ParA-ATP bound to it at site i with number 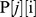 (≥0), 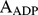: cytoplasmic ParA-ADP with number AADP (≥0), 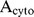: cytoplasmic ParA-ATP with number ACYTO (≥0) The reactions and corresponding propensities 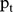 are described in Table 1. Parameter values used are listed in Table 2.

We varied the exact number of ParA-ATP molecules forming a complex that are required to completely immobilize the plasmid and this variation does not alter the qualitative behavior of the system. Introduction of a low spontaneous ParA-ATP hydrolysis parameter k_off_ also does not alter the behaviour of the system. We do not keep track of the spatial positions of ParA-ADP and ParA-ATP in the cytoplasm. Instead we merely keep track of their number.

The ParA concentration is assumed to be constant throughout the cell cycle, consistent with the total ParA-GFP fluorescence as a function of cell volume when expressed from an inducible promoter (S1C Fig.). In accordance with estimates for average ParA copy numbers obtained by semi-quantitative Western blots (S1B Fig.), the ParA concentration is assumed to be 2400 ParA (dimers) per µm of nucleoid. Simulations start at time t = 0 and run until time t, updated according to the Gillespie algorithm, exceeds a predefined time T. To simulate nucleoid growth during the cell cycle the nucleoid lattice is extended by two sites of size dx (not containing any ParA or plasmids), at one randomly chosen position along the nucleoid length. Such a growth event occurs at regular time intervals. Reaction propensities are then updated in accordance with the new state.

In Fig. 2B the nucleoid grows from 1.5 µm to 3 µm in T = 40 min, reflecting one cell cycle. Initially a quarter of the total ParA in the system is in the cytoplasmic ParA-ADP form, 11 ParA-ATP are bound to each plasmid to ensure initial anchoring, and the rest are bound randomly to the nucleoid. In Fig. 2B the plasmid is initially located at site 0. In the simulations used to generate the histograms shown in Fig. 2C, S3A, all plasmids are initially distributed randomly across the nucleoid. At regular time intervals of 5 s the simulation state is output along with the plasmid positions to generate a time-averaged probability distribution for the plasmid positions along the long axis of the cell. In cases where the total number of plasmids (n_p_) is more than one, the plasmids are ordered and labeled 1…n_p_ according to their positions (by increasing site number) along the nucleoid. Their position is then used to generate distributions for every plasmid label 1…n_p_ for that particular overall number of plasmids n_p_.

In the event of plasmid duplication at a particular site where an existing plasmid is located, a new plasmid without any bound ParA is added to the same site and the reaction propensities are updated accordingly. In case of two or more existing plasmids, one is chosen randomly for duplication. Plasmid duplication events in Fig. 2B occur at regular time intervals T/3, although the model behaves equally well with duplication at any time as it dynamically segregates the plasmids to equally spaced positions.

### Directed motion model

The nucleoid was represented as a rectangular lattice divided into square sites of sides dx = 5 nm. The long axis could grow from 1.5 µm to 3 µm in length, while the short axis of the nucleoid lattice remained fixed. For wild-type directed motion model simulations the short axis length was 30 nm (directed motion model with short polymers) and 25 nm (directed motion model with long polymers). Thus every site had a coordinate along the long axis (labelled as 0… L-1) as well as a coordinate along the short axis (labelled 0… S-1). Reactants are: 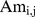: mobile ParA-ATP at site (i,j) with number 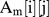 (≥0), 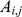: polymeric ParA-ATP at site (i,j) with number 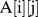 (0 or 1); 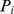: plasmids at site i with number 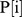 (≥0); 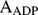: cytoplasmic ParA-ADP with number 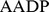 (≥0) and 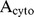: cytoplasmic ParA-ATP with number 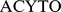 (≥0). The reactions and corresponding propensities 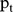 are listed in Table 3. Parameter values used are listed in Table 4.

In the perturbed-nucleoid simulations, mobile ParA-ATP can diffuse past a plasmid with 10% (short) or 100% (long) of the normal diffusion rate and the short axis length of the nucleoid is altered to 10 nm in the long polymer model. Lastly, to allow for mobile ParA-ATP to move past the plasmid without being hydrolyzed, k_mB_ is reduced 10-fold compared to its standard value.

As for the diffusion/immobilization model, the total ParA concentration was constrained to be 2400 ParA (dimers) per µm of nucleoid (long axis) and the total length of simulated time was T = 40 min. Initially a quarter of the total number of ParA in the system was in the cytoplasmic ParA-ADP form, with the rest distributed randomly on the nucleoid in the mobile ParA-ATP form. Initial plasmid positioning, state output, plasmid position distribution generation and plasmid duplication rules were also as described previously. Nucleoid growth was implemented as described previously, with one generalization: a position along the long axis of the nucleoid was first chosen randomly. Then two nucleoid slices of 1 site (along the long axis) by S sites (along the short axis) were inserted.

### Plasmids and strains

The ParA-GFP fusion and *tetO-*TetR-mCherry plasmid labeling system were described previously [9], [12]. To obtain the functional ParB-GFP fusion, the *parB* gene in the *par2* locus was replaced by *parB::sfGFP* and inserted in a mini-R1 test-plasmid. See S2 Text for more details on the strains and plasmids construction, semi-quantitative ParA western blotting and supplemental figure data analysis.

### Epifluorescence microscopy

*E. coli* strains carrying plasmids of interest (see Table S1, S2 in S2 Text for details on strains and plasmids) were grown to stationary phase while being shaken at 37°C in LB medium supplemented with appropriate antibiotics (30 or 50 µg/ml ampicillin, 25 or 50 µg/ml kanamycin, 15 µg/ml chloramphenicol), with the exception of the *muk* strains, which were grown at 24°C. Cultures were diluted to an OD_450_ of 0.025 in antibiotic-free M9 minimal medium containing supplements (0.2% casamino acids, 0.2% glycerol, 1 µg/ml thiamine, 1 mM MgSO_4_, 0.1 mM CaCl_2_). Inoculated cultures were incubated until an OD_450_ of ≈0.2 was reached, typically taking 3 h.

When nalidixic acid was used to condense the nucleoids, the antibiotic was added to a growing culture at a final concentration of 50 µg/ml two hours before imaging. Where appropriate, culture samples were mixed with Hoechst 33342 (Invitrogen) at a final concentration of 50 µg/ml for DNA staining immediately before microscopy.

For imaging, cells were immobilized on 1.5% agarose-M9 pads mounted on microscopy slides using Gene Frames (Thermo Scientific). All microscopy experiments, unless specified otherwise (see below), were carried out using an Olympus IX71 inverted microscope with a CoolSNAP HQ EMCCD digital camera (Photometrics, pixel size  = 0.066 µm). A temperature-controlled incubation chamber (Applied Scientific) fitted to a Weather Station (Precision Control) kept samples at a constant 30°C. Images were acquired using SoftWoRx version 5.5.0 with a Zeiss Plan-Neofluar 100X/1.30 NA oil objective and Olympus Mercury 100 W burner (U-LH100HG) fluorescent light source. Filter set specifics are given in Table S3 in S2 Text.

### Optical sectioning of fluorescence signals from ParA-GFP and Hoechst-stained nucleoid DNA

Expression of ParA-GFP from plasmid pGE230 (mini-R1, *par^-^*, *P_lac_::parA::eGFP*) in *E. coli* strain KG22 or FS1 (KG22Δ*matP*) was induced by adding 10 µM IPTG to the culture medium two hours before microscopy. A 31 image Z-stack with 0.1 µm section widths was taken for all projections (exposure times Phase Contrast (GFP channel): 0.05 s, ParA-GFP: 1.5 s, Phase Contrast (Hoechst channel): 0.1 s, Hoechst: 2 s). Image stacks were subsequently deconvolved using SoftWoRx v.5.5.0 with the following parameters: 10 iterations, medium noise reduction, conservative method.

### Measuring asymmetry in ParA-GFP distributions using optical sectioning

Expression of ParA-GFP from plasmid pSR233 (mini-R1, *par2^+^*, *P_lac_::parA::eGFP, tetO120*) in *E. coli* KG22 cells harboring pSR124 (*P_BAD_::tetR::mCherry*) was induced by adding 10 µM IPTG to the culture medium one hour before microscopy. Samples were treated with Hoechst stain and imaged immediately thereafter. Expression of TetR-mCherry was not induced, as baseline activity of P_BAD_ produced sufficient amounts of TetR-mCherry to detect foci in a single image at mid Z-height. Similarly, a single Hoechst stain image was acquired. For ParA-GFP, a 21 image Z-stack with 0.2 µm section widths was taken (exposure times Phase Contrast: 0.1 s, TetR-mCherry 1.5 s, ParA-GFP: 1.5 s, Hoechst 0.15 s). Images were acquired using a Zeiss Axiovert 200 M inverted epifluorescence microscope with a Zeiss Plan-Neofluar 100X/1.3 NA oil objective in a temperature-controlled room at 22°C. The microscope was controlled using MetaMorph software version 7.7.5.0 (Molecular Devices, Inc.). Cells were illuminated using a Lambda LS xenon-arc lamp and images acquired using a CoolSnap HQ^2^ EMCCD digital camera (Photometrics, pixel size  = 0.0625 µm). Filter set specifics are given in Table S3 in S2 Text.

### Time-lapse imaging of plasmid foci movement

Plasmid foci of the *par* ^-^ mini-R1 plasmids pMH82tetO120 (*par* ^-^, *tetO120* ^+^) or pSR236 (*parC1* ^+^, Δ*parA*, *parB* ^+^, *parC2* ^+^, *P_lac_::parA::eGFP*, *tetO120* ^+^) in *E. coli* strain SR1 (KG22Δ*pcnB*) were visualised by labelling *tetO* arrays on the plasmid *in trans* with TetR-mCherry provided from the pSR124 vector (see [40] for the original method). TetR-mCherry expression was induced by adding L-arabinose to a final concentration of 0.02% to growing cultures for 15 minutes, followed by catabolite repression with 1% glucose for 10 minutes. In strains harbouring pSR236, expression of ParA-GFP was induced by the addition of 10 µM IPTG inducer 2 h before microscopy. Time-lapse image series were acquired for different total durations/time intervals: 1 min/4 s or 15 min/30 s for pMH82tetO120 (exposure times phase contrast: 0.1 s; TetR-mCherry: 1.5 s) and 1 min/5 s or 15 min/20 s for pSR236 respectively (exposure times phase contrast: 0.1 s; TetR-mCherry: 1.5 s; ParA-GFP: 1 s). The maximum rate of image acquisition possible with our imaging system was every 4 s and 5 s (without and with ParA-GFP channel) for pMH82tetO120 and pSR236 respectively. Sample focus was maintained in the mid-cell plane throughout the experiment using the UltimateFocus system (Applied Precision) sampling and refreshing before the acquisition of each individual frame.

### Plasmid focus positioning microscopy

*E. coli* strains KG22, FS1 (KG22Δ*matP*), FS2 (KG22: *mukE::kan*) or FS3 (KG22: *mukF::kan*) harbouring pFS21 (mini-R1, *parC1^+^*, *parA^+^*, *parB::sfGFP*, *parC2^+^*) were grown to an OD_450_ of 0.3. Samples were treated with Hoechst stain and imaged immediately in the mid-cell plane (exposure times ParB-GFP: 1 s, Hoechst: 0.5 s). Images of *muk* strains were acquired using a Zeiss Axiovert 200 M inverted epifluorescence microscope with a Zeiss Plan-Neofluar 100X/1.3 NA oil objective in a temperature-controlled room at 22°C. The microscope was controlled using the MetaMorph software version 7.7.5.0 (Molecular Devices, Inc.). Cells were illuminated using a Lambda LS xenon-arc lamp and images acquired using a CoolSnap HQ^2^ EMCCD digital camera (Photometrics, pixel size  = 0.0625 µm). Filter set specifics are given in Table S3. Other strains were imaged using both the Olympus IX71 and Zeiss Axiovert 200 M systems described above.

### Plasmid foci mobility determination

Using the MATLAB-based software suite MicrobeTracker (MT) [27], we determined *E. coli* cell outlines from phase contrast (PC) images, as well as the distribution of *tetO-*TetR-mCherry-labeled plasmids along the long axis of cells. The cell outlines were used together with the MATLAB tools spotFinderZ and spotFinderM [27] to determine *tetO-*TetR-mCherry foci positions in *par^-^* time-lapses of 1 min (short) or 15 min (long) in duration with images taken at intervals of 4 s or 30 s respectively. The linear *tetO-*TetR-mCherry distribution was used to control the peak detection method for false positives/negatives. For the short time-lapses we analysed cells with one or more foci, although all our results were unchanged if analysis was restricted to one focus cells to prevent potential foci labelling errors. For the long time-lapses, we only analysed cells exhibiting one focus. This was due to difficulties in distinguishing between multiple foci due to merging/splitting events, out of focus plane movement and photobleaching when acquiring images using a time interval of 30 s. These effects could have resulted in biases in the analysis due to labelling errors. We were unable to lower the time interval and simultaneously image for long time periods due to TetR-mCherry photobleaching.

At every time point the two-dimensional squared foci displacements r^2^(τ) after time lag τ were determined. All measured displacements for the same time lag were then averaged together to obtain Mean Square Displacements (MSD) 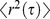 with time lags from 4 s to 15 min (Fig. 3B,C,S3B). The measured plasmid displacement r_p_(τ) can report the true plasmid displacement r_p_(τ) at a resolution no greater than our measurement error, which can be up to 0.1 µm due to microscope drift. Our measurements are also limited by a finite pixel size of 0.066 µm. We therefore have: 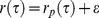, where ε is the error due to both of the above effects. Squaring and averaging over many plasmid trajectories results in an MSD: 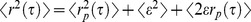. The last term vanishes due to averaging, but the second term remains and generates a small time independent value for τ>0. Even at short timescales of up to a minute, the MSD has a nonlinear shape, as has been reported before [26]. This is fully consistent with subdiffusive motion on these timescales. We thus expect the experimentally observed planar MSD for free particle subdiffusion in three dimensions to have the form: 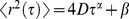. We carefully measured the *par^-^* MSD up to 1 min with short time intervals between measurements (Fig. 3B). We performed a nonlinear least squares fit (weighted by the standard error of the mean (SEM): 1/SEM(τ)) for 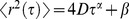 resulting in the values α = 0.78±0.04, D = 6.8±1.2×10^−4^ µm^2^s^−α^, β = 6±1×10^−3^ µm^2^ (R^2^ = 0.99, p-values: 4×10^−10^, 1×10^−4^ and 8×10^−4^ respectively). On longer timescales up to 15 min (Fig. 3C), plasmid mobility also showed subdiffusive behaviour with a similar analysis giving α = 0.78±0.05, D = 6.2±2.1×10^−4^ µm^2^s^−α^, β = 4±1×10^−2^ µm^2^ (R^2^ = 0.99, p-values: 8×10^−15^, 8×10^−3^ and 2×10^−3^ respectively). Analysing the two datasets combined (Fig. 3C) also generated consistent results, although the constant β was not significantly different from zero in this case: α = 0.73±0.02, D = 9.7±1.3×10^−4^ µm^2^s^−α^, β = 1.6±2.4×10^−3^ µm^2^, (R^2^ = 0.99, p-values: 8×10^−15^, 8×10^−3^ and 0.50 respectively, fit shown in Fig. 3C). Fitting 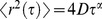 instead to this combined data set did not alter our estimates for α and D significantly. On all observable timescales (i.e. 4 s and longer) the experimentally found *par^-^* MSD is bounded from above by the function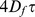, with D_f_  =  10×10^−4^ µm^2^s^−1^. Moreover, free diffusion with diffusion constant D_f_ inside a box of cellular dimensions still exceeds the experimental subdiffusive mobility.

### Determining plasmid foci positions

Cell outlines and linear projections of ParB-GFP and Hoechst signal distributions along their long axis were determined as described above using MicrobeTracker (MT) [27]. ParB-GFP foci detection of snapshots was also performed using the methods described above. The positions of the half-maxima of the linear Hoechst signal distribution in every cell were then determined. We defined the nucleoid length as the length between the two half-maxima of the Hoechst stain. This analysis allowed us to determine the positions of plasmid foci with respect to the nucleoid.

### ParA asymmetry analysis

Here, we summed 6 planes that are in focus from a Z-stack of ParA-GFP fluorescence signal images (dz = 0.2 µm), although the results are not different when using the ParA-GFP signal obtained from single confocal planes focused at mid-cell. Cell outlines, linear projections of ParA-GFP, *tetO-*TetR-mCherry and Hoechst stain fluorescence signal distributions, and *tetO-*TetR-mCherry foci positions were determined as described above. We confirmed that positioning of the *tetO*-TetR-mCherry foci from this dataset was similar to that measured previously [9]. In cells containing one plasmid focus (n = 134), the ParA-GFP fluorescence signal from pole to plasmid position was summed and divided by the respective pole-to-plasmid distance. This generates two ParA-GFP fluorescence densities I_L_ and I_R_ for either side extending to the two cell poles. This allows us to compute the normalized ParA asymmetry measure |I_L_-I_R_|/|I_L_+I_R_|. Irrespective of the plasmid position, a completely uniform fluorescence distribution would give an asymmetry value of zero. On the other hand, if all the ParA-GFP was located on one side of the plasmid the asymmetry measure would be one. Using a single confocal plane focused at mid-cell, we also computed the Hoechst asymmetry measure with respect to the plasmid position in the same manner.

As shown in [27] by using the same MT software package for analysis, the MinD-YFP asymmetry measure with respect to mid-cell follows an approximate sinusoidal oscillation over time, with a cell-length-dependent oscillation amplitude. In large cells the MinD-YFP oscillations are clearest with an amplitude 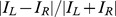 of around 0.6. To generate an asymmetry measure appropriate for the MinD-YFP oscillations, we sampled 10^3^ time points t uniformly in [0,2π] (which constitutes one period). We then computed for every time point 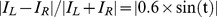. The resulting asymmetry distribution (Fig. 5B) therefore reflects the experimental MinD-YFP asymmetry with respect to mid-cell in large cells [27]. In this way, we can directly compare the asymmetry present in the ParA-GFP and Hoechst signal distributions with that induced by the spatiotemporal oscillations of MinD-YFP. We also generated asymmetry measures using our directed motion model. In simulation outcomes shown in Fig. 4B (directed motion model with short polymers) and S4B (directed motion model with long polymers), the plasmid position, cytoplasmic ParA-ADP, cytoplasmic ParA-ATP, nucleoid-bound mobile ParA-ATP and polymeric ParA-ATP levels on either side of the plasmid were output at regular time intervals of dt = 5 s during a time period prior to plasmid duplication (first 2 min and 1.5 min of simulated time for directed motion model with short and long polymers respectively). Cytoplasmic ParA was assumed to be uniformly distributed throughout the cell (independently of the plasmid position), thus effectively only contributing to the denominator |*I_L_ + I_R_*|. With this information we computed the ParA asymmetry using the same method as described for the experimental data. Results are shown in Fig. 5B (short polymers) and S5B Fig. (long polymers). It should be noted that according to both models, the ParA asymmetry remains very low once a plasmid is stably positioned at mid-cell, pushing the asymmetry distribution further towards zero over time. This is consistent with time lapses where stable equally spaced plasmid foci positioning correlates with ParA-GFP on either side of a plasmid focus (Fig. 5C and [9]).

### Three dimensional nucleoid and ParA structure analysis

To compare the extent of overlay and 3D structure of Hoechst (nucleoid DNA) stain and ParA-GFP, we first had to align the Z-stack pairs in an unbiased manner. To achieve this, one phase contrast (PC) image (at mid z height) of the Hoechst signal sections was aligned with one GFP section PC image (at the same z position) using the TurboReg ImageJ plugin (option: translation) [41], after cropping both PC images to match the output size of the deconvolved Z-stacks. Using the same translation as for the Hoechst PC image, Hoechst Z-stacks were then translated in ImageJ to align them with the ParA-GFP Z-stacks.

We determined cell outlines in MT as described above using the PC image acquired with the GFP channel and excluded cells that did not show visible ParA-GFP and Hoechst stain simultaneously. We then computed the linear distributions (for every z height) along the long cell axis for the deconvolved Hoechst and ParA-GFP Z-stacks. We next determined for the ParA-GFP and Hoechst signals separately in every cell the maximal intensity value in the whole cell (I_maxcell_) and the maximal values at every z height (I_max_(z)). To find the 9 z planes from the Z-stacks (dz = 0.1 µm) that are in-focus for each cell in an automated fashion, we summed I_max_(z) over 9 consecutive z positions including a given starting plane and determined the starting plane that gave the largest associated summed value. This starting plane and its 8 consecutive planes formed the in focus plane set. We verified that this method generated the right focus planes by inspecting the chosen planes visually for several cells. This method circumvents the problem of different focus planes for cells on the same image stack as well as alignment inaccuracies in the z direction between ParA-GFP and Hoechst signals which are difficult to control for manually.

Visual comparison of the nucleoid shape between WT and nalidixic acid (Nal) treated cells (Fig. 6A,B, S6A) revealed clear differences. In Nal-treated cells, the nucleoid signals, where present inside a cell, were more uniform along the long cell axis than in the WT (S6A,B Fig.). Shape differences were also visible in the raw Z-stacks suggesting they were not artefacts of the deconvolution method. To quantify these shape differences in an unbiased and systematic manner, we performed the following analysis (S6B Fig.).

We reasoned that a more uniform pattern would result in a profile along the long axis that resembled a first harmonic (first non-constant term of a Fourier expansion) between the nucleoid edges. Such a harmonic would not fit so well to a more spatially oscillating pattern that would arise, for example, from helical structures. Using the Hoechst stain I_maxcell_ and the I_max_(z) arising from the 9 relevant focus planes we determined the half-maximum intensity locations along the long cell axis closest to the cell poles x_L_ and x_R_ at every z height. At every focus plane z height we could now define the ‘first harmonic’ function defined for x_L_≤x≤x_R_:



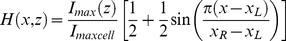
.

For every (x,z) we calculated the squared error SE(x,z) between the actual intensity value I(x,z) and H(x,z): 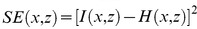. Lastly we summed over the SEs at every (x,z) and divided by the number of position points (x,z) to obtain a single measure of deviation SE_cell_ in a cell that is independent of the number of data points (and thus nucleoid size) and expression level variation between cells (because of normalization to I_maxcell_). We then performed a Wilcoxon rank sum test on the set of SE_cell_ comparing a population of WT cells with nucleoid-perturbed cells (n_WT_ = 678 and n_Nal_ = 862). Nucleoid shapes in Nal-treated cells were indeed altered (p<10^-149^). Note that this method did not detect a notable shape change in *matP* cells (n*_matP_* = 579), potentially due to our techniques not being sufficiently sensitive.

To quantitate the colocalization of ParA-GFP and Hoechst signal in each cell, we also calculated, for every cell, the Pearson's correlation coefficient r_P_ using all the intensity values I_ParA-GFP_(x,z) and I_Hoechst_(x,z) [42].

To determine the fraction of ParA-GFP intensity signal that overlaps with Hoechst signal and vice versa we computed Manders overlap coefficients [42]. This method requires a choice of threshold T_Manders_ to distinguish between positions (x,z) that are considered to contain or lack sufficient intensity signal. We therefore performed our analyses for the complete range of threshold values to show that our qualitative conclusions are insensitive to the choice of a particular T_Manders_ (Fig. 6C, 7C). Manders overlap coefficients of ParA-GFP and Hoechst were calculated as follows:
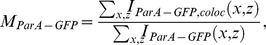
with 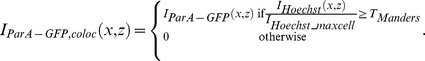



Likewise the Manders overlap coefficient of Hoechst onto ParA-GFP is defined as:
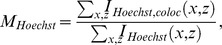
with
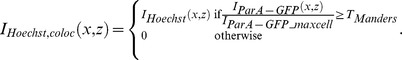



Note that taking T_Manders_ = 0, will generate an overlap coefficient of one by construction. The normalization to I_maxcell_ in determining the colocalizing positions allows the overlap coefficients to be comparable between cells.

In a small fraction of cells the alignment procedure described above did not result in proper alignment. This is clearly reflected in the r_P_ values being considerably lower for these cases than for the cell population mean r_P_ value. However, without excluding these few, possibly false negative, cases the population mean r_P_ value is still high (0.81 and 0.68 for WT and Nal-treated cells respectively), indicating that ParA-GFP and Hoechst signals generally correlate strongly at a population level. Poor alignment affects Manders overlap coefficients for the ParA-GFP and Hoechst signals on average equally and is not biased towards a particular strain/treatment. Therefore the observed misalignment of a small fraction of cells does not affect the qualitative conclusions that we state in this study.

Note that in *matP* cells, we did not observe any significant alteration in intensity correlation (r_P_ = 0.80 for *matP*), nor ParA-GFP overlap coefficient, as compared to the WT. This result was expected given that we could not detect any significant nucleoid structure alteration, as described above.

## Supporting Information

S1 Fig *par2* protein functionality and expression levels. (**A**) Plasmid loss-frequency assay showing pFS21 stabilisation to wild-type levels by the recombinant *par2* locus encoding *parB::sfGFP*, confirming functionality of the fluorescent fusion protein. Plasmids used are pRBJ200 (*par* ^-^, red), pFS19 (*par2* ^+^, black) and experimental vector pFS21 (*parC1^+^*, *parA^+^*, *parB::sfGFP*, *parC2* ^+^, green), n = 2, error bars: standard error of the mean. (**B**) Representative section of semi-quantitative Western blot used for approximating ParA molecule numbers *in vivo*. Cell lysate samples of strain KG22 carrying a mini-R1 plasmid lacking (pRBJ200) or containing *par2* (pGE2) were compared to plasmid-free KG22 cell lysate mixed with known amounts of purified _His6_ParA. Standard curve generated from intensity measurements from this blot has R^2^ = 0.965. Band intensities were measured and quantified using the ImageQuant TL 1D Gel Analysis Software, (n = 3). (**C**) Scatter plot of ParA-GFP total fluorescence signal in single WT cells as a function of cell volume, when expressed from an inducible promoter (*P_lac_*). The different color labels indicate the number of plasmid foci. Plasmids: pSR233 (mini-R1, *par2^+^*, *P_lac_::parA::eGFP, tetO120*) and pSR124 (*P_BAD_::tetR::mCherry*). (**D**) Scatter plot of ParB-GFP total fluorescence signal in single WT cells as a function of cell volume, when expressed from its native promoter. Plasmid: pFS21 (*parC1^+^*, *parA^+^*, *parB::sfGFP*, *parC2* ^+^); color labeling as in (**C**).
(PDF)

S2 Fig Plasmid foci are equally spaced over the nucleoid irrespective of nucleoid length or plasmid focus copy number. (**A**) Scatter plot of plasmid foci positions (blue, green, red, cyan) with respect to nucleoid edges (purple) and cell edges (black) for wild-type cells. Strains and plasmids used for S2 Fig. are as described in Fig. 1. (**B**) Histograms of plasmid foci positions shown in (**A**) relative to nucleoid length.
(PDF)

S3 Fig Diffusion/immobilization model can move and maintain plasmids at equally spaced positions. (**A**) Time-averaged plasmid position distributions for diffusion/immobilization model with n_p_ = 3,4 on a simulated nucleoid growing from 1.5 µm to 3 µm in 40 min without plasmid duplication. Plasmid distributions were obtained by sampling positions every 5 s in 36 independent simulations. (**B**) Plots as in Fig. 3B except with experimental *par^-^* (red, green, blue) plasmid trajectories in which plasmid location is within a region of normalized Hoechst stain intensity I equal to or higher than the values indicated in the legend. The corresponding plasmid copy numbers (n_par2+_ = 763, n_par-, I≥0_ = 747, n_par-, I≥0.5_ = 592, n_par-, I≥0.75_ = 401) indicate that a large fraction of *par^-^* plasmids do indeed reside in the nucleoid region; error bars: standard error of the mean. (**C**) Plots of 13 segregation events of *par2^+^* pSR236 (mini-R1, *parC1^+^*, *parA^-^*, *parB^+^*, *parC2^+^, tetO120*, *P_lac_::parA::eGFP*) plasmids in *E. coli* cells harboring pSR124 (*P_BAD_::tetR::mCherry*). Shown is the additionally segregated distance (colored lines) as a function of time, both with respect to the start of each segregation event. A segregation event is defined as two foci that are initially ≤0.3 µm apart and subsequently segregate ≥0.8 µm further apart within 20 s. The horizontal line (black) indicates 0.8 µm.
(PDF)

S4 Fig The directed motion model can equally space plasmids over the nucleoid, and is not critically dependent on the extent of ParA polymerization. (**A**) Time-averaged plasmid position distributions for directed motion model with short polymers with n_p_ = 3,4 plasmids on a simulated nucleoid growing from 1.5 µm to 3 µm in 40 min without plasmid duplication. Plasmid distributions were obtained by sampling positions every 5 s in 36 independent simulations. (**B**) Typical simulation kymograph of the directed motion model with long polymers. Long polymers extend from nucleoid ends in a growing cell, where plasmid (red) is initially directed from a nucleoid edge to mid-cell by ParA (green) filament competition. After plasmid duplication, the system dynamically self-organizes to reacquire equal plasmid spacing. (**C**) Time-averaged plasmid position distributions for directed motion model with long polymers with n_p_ = 1–4 plasmids. Simulated nucleoid growth and plasmid distributions obtained as in (**A**).
(PDF)

S5 Fig Hoechst DNA stain and ParA-GFP signal asymmetry are relatively low and uncorrelated to plasmid focus positioning. (**A**) Scatter plot of ParA-GFP asymmetry measure as a function of cell length (n = 134). (**B**) ParA asymmetry prediction from the directed motion model with long polymers. Comparison shown to experimental ParA-GFP (n = 134), Hoechst (n = 134) and MinD-YFP distributions [7]. (**C**) Scatter plot of ParA-GFP and Hoechst asymmetry as a function of (a single) plasmid focus position relative to cell length.
(PDF)

S6 Fig Correlation between Hoechst and ParA-GFP distributions. (**A**) Normalized fluorescence intensity profiles along the long cell axis for 9 in focus z heights (dz = 0.1 µm) resulting from deconvolved Z-stacks in representative WT and Nal-treated strains. Many cases (representative examples shown) support the existence of linear ParA-GFP structures, although the inherent optical resolution of the imaging prohibits stronger conclusions about the presence or absence of narrow linear ParA-GFP filaments. For every cell having detectable Hoechst and ParA-GFP signals, the corresponding profiles were used for the systematic colocalization analyses. (**B**) Graphical illustration of the unbiased systematic ‘first harmonic’ analysis of deconvolved 3d Hoechst signal inside representative cells in WT and Nal-treated strains. The Hoechst (blue) profiles indicate the signal intensities (integrated over the cell width) along the long cell axis at 9 in focus z heights with corresponding ‘first harmonics’ (dotted red curves, see Materials and Methods). Fluorescence signal distributions deviate significantly more from the first harmonics in WT compared to Nal-treated cells (Wilcoxon rank sum test, p<10^−149^), showing that Hoechst DNA stain distributions are perturbed in the latter. This analysis is independent of nucleoid length, which is altered in Nal-treated strains as compared to WT (S7A Fig.).
(PDF)

S7 Fig Comparison of plasmid foci position histograms in cells with perturbed nucleoid morphology to completely randomized plasmid distributions. (**A**) Mean nucleoid length (error bars: standard error of the mean) of cells used for the plasmid positioning analysis shown in Figs. 1B, S2A and (**B,C**) in different strains: WT (n = 1695), *mukE* (n = 1378), *mukF* (n = 1555), *matP* mutants (n = 2995) and cells treated with nalidixic acid (Nal) (n = 1127). According to unpaired *t* tests, all mutants and Nal show a mean differing from WT (p<10^−3^). Although the average nucleoid length in *matP* mutants decreased, the average number of nucleoids per cell increased compared to WT (p<10^−41^) due to a large fraction of cells exhibiting 2 nucleoids (using our half maximum criteria). This observation is consistent with the previously proposed function of MatP in preventing early segregation of duplicated Ter macrodomains. (**B**) Scatter plot of n_p_ = 1–4 plasmid foci positions (blue, green, red, cyan) with respect to nucleoid edges (purple) and cell edges (black) for *mukE*, *mukF* mutant cells. (**C**) As in (**B**) for *matP* mutants and cells treated with 50 µg/ml nalidixic acid (Nal). (**D**) Histograms of n_p_ = 3,4 plasmid foci positions shown in (**B,C**) relative to nucleoid size. (**E**) Histograms of 10^5^ datasets for each of n_p_ = 1–4, where for each dataset plasmids are positioned in [0,100] with a uniform distribution, independent from each other and consequently labeled 1..n_p_ according to their position. This protocol induces an inherent spatial ordering. By comparing these distributions with the WT experimental data shown in Fig. 1C (n_p_ = 1,2) and S2B Fig. (n_p_ = 3,4) it is clear that the *parABC* system positions plasmid foci much more precisely, although the effect of active positioning becomes less clear as n_p_ increases. (**F**) Time-averaged plasmid position distributions for directed motion model with short and long polymers for n_p_ = 3–4 (short) and n_p_ = 1–4 (long) on simulated growing nucleoids without plasmid duplication. Results obtained from 124 independent simulations, where ParA-ATP could now diffuse past a plasmid (see Materials and Methods).
(PDF)

S1 Text Supplementary text to section: Mathematical analysis shows that dynamic ParA concentrations can generate equal plasmid spacing. This text contains the derivation that in our mathematical model a symmetric ParA concentration implies equal plasmid spacing in case of ParB-*parC*-mediated ParA-ATP hydrolysis with any rate *k_B_*.
(PDF)

S2 Text Supplementary materials and methods including Table S1, S2 and S3.
(DOCX)

## References

[1] Gerdes K, Howard M, Szardenings F (2010) Pushing and Pulling in Prokaryotic DNA Segregation. Cell 141: 927–942. 20550930 10.1016/j.cell.2010.05.033

[2] Gayathri P, Fujii T, Moller-Jensen J, van den Ent F, Namba K, et al (2012) A Bipolar Spindle of Antiparallel ParM Filaments Drives Bacterial Plasmid Segregation. Science 338: 1334–1337. 23112295 10.1126/science.1229091 PMC3694215

[3] Fogel MA, Waldor MK (2006) A dynamic, mitotic-like mechanism for bacterial chromosome segregation. Genes Dev 20: 3269–3282. 17158745 10.1101/gad.1496506 PMC1686604

[4] Ptacin JL, Lee SF, Garner EC, Toro E, Eckart M, et al (2010) A spindle-like apparatus guides bacterial chromosome segregation. Nat Cell Biol 12: 791–U746. 20657594 10.1038/ncb2083 PMC3205914

[5] Derome A, Hoischen C, Bussiek M, Grady R, Adamczyk M, et al (2008) Centromere anatomy in the multidrug-resistant pathogen Enterococcus faecium. Proc Natl Acad Sci USA 105: 2151–2156. 18245388 10.1073/pnas.0704681105 PMC2538891

[6] Roberts MAJ, Wadhams GH, Hadfield KA, Tickner S, Armitage JP (2012) ParA-like protein uses nonspecific chromosomal DNA binding to partition protein complexes. Proc Natl Acad Sci USA 109: 6698–6703. 22496588 10.1073/pnas.1114000109 PMC3340030

[7] Savage DF, Afonso B, Chen AH, Silver PA (2010) Spatially Ordered Dynamics of the Bacterial Carbon Fixation Machinery. Science 327: 1258–1261. 20203050 10.1126/science.1186090

[8] Vecchiarelli AG, Han YW, Tan X, Mizuuchi M, Ghirlando R, et al (2010) ATP control of dynamic P1 ParA-DNA interactions: a key role for the nucleoid in plasmid partition. Mol Microbiol 78: 78–91. 20659294 10.1111/j.1365-2958.2010.07314.x PMC2950902

[9] Ringgaard S, van Zon J, Howard M, Gerdes K (2009) Movement and equipositioning of plasmids by ParA filament disassembly. Proc Natl Acad Sci USA 106: 19369–19374. 19906997 10.1073/pnas.0908347106 PMC2775997

[10] Ringgaard S, Ebersbach G, Borch J, Gerdes K (2007) Regulatory cross-talk in the double par locus of plasmid pB171E. J Biol Chem 282: 3134–3145. 17092933 10.1074/jbc.M609092200

[11] Ringgaard S, Lowe J, Gerdes K (2007) Centromere pairing by a plasmid-encoded type I ParB protein. J Biol Chem 282: 28216–28225. 17644524 10.1074/jbc.M703733200

[12] Ebersbach G, Gerdes K (2001) The double par locus of virulence factor pB171: DNA segregation is correlated with oscillation of ParA. Proc Natl Acad Sci USA 98: 15078–15083. 11752455 10.1073/pnas.261569598 PMC64986

[13] Ebersbach G, Gerdes K (2004) Bacterial mitosis: partitioning protein ParA oscillates in spiral-shaped structures and positions plasmids at mid-cell. Mol Microbiol 52: 385–398. 15066028 10.1111/j.1365-2958.2004.04002.x

[14] Ebersbach G, Ringgaard S, Moller-Jensen J, Wang Q, Sherratt DJ, et al (2006) Regular cellular distribution of plasmids by oscillating and filament-forming ParA ATPase of plasmid pB171. Mol Microbiol 61: 1428–1442. 16899080 10.1111/j.1365-2958.2006.05322.x

[15] Sengupta M, Nielsen HJ, Youngren B, Austin S (2010) P1 Plasmid Segregation: Accurate Redistribution by Dynamic Plasmid Pairing and Separation. J Bacteriol 192: 1175–1183. 19897644 10.1128/JB.01245-09 PMC2820840

[16] Hatano T, Niki H (2010) Partitioning of P1 plasmids by gradual distribution of the ATPase ParA. Mol Microbiol 78: 1182–1198. 21091504 10.1111/j.1365-2958.2010.07398.x

[17] Hwang LC, Vecchiarelli AG, Han YW, Mizuuchi M, Harada Y, et al (2013) ParA-mediated plasmid partition driven by protein pattern self-organization. EMBO J 32: 1238–1249. 23443047 10.1038/emboj.2013.34 PMC3642677

[18] Vecchiarelli AG, Hwang LC, Mizuuchi K (2013) Cell-free study of F plasmid partition provides evidence for cargo transport by a diffusion-ratchet mechanism. Proc Natl Acad Sci USA 110: E1390–E1397. 23479605 10.1073/pnas.1302745110 PMC3625265

[19] Sugawara T, Kaneko K (2011) Chemophoresis as a driving force for intracellular organization: Theory and application to plasmid partitioning. Biophys Soc Japan 7: 77–88. 10.2142/biophysics.7.77 PMC5036777 27857595

[20] Vecchiarelli AG, Neuman KC, Mizuuchi K (2014) A propagating ATPase gradient drives transport of surface-confined cellular cargo. Proc Natl Acad Sci USA 111: 4880–4885. 24567408 10.1073/pnas.1401025111 PMC3977271

[21] Lim HC, Surovtsev IV, Beltran BG, Huang F, Bewersdorf J, et al (2014) Evidence for a DNA-relay mechanism in ParABS-mediated chromosome segregation. Elife 3: e02758. 24859756 10.7554/eLife.02758 PMC4067530

[22] Slepoy A, Thompson AP, Plimpton SJ (2008) A constant-time kinetic Monte Carlo algorithm for simulation of large biochemical reaction networks. J Chem Phys 128: 205101. 18513044 10.1063/1.2919546

[23] Parry BR, Surovtsev IV, Cabeen MT, O'Hem CS, Dufresne ER, et al (2014) The Bacterial Cytoplasm Has Glass-like Properties and Is Fluidized by Metabolic Activity. Cell 156: 183–194. 24361104 10.1016/j.cell.2013.11.028 PMC3956598

[24] Weber SC, Spakowitz AJ, Theriot JA (2010) Bacterial Chromosomal Loci Move Subdiffusively through a Viscoelastic Cytoplasm. Phys Rev Lett 104: 238102. 20867274 10.1103/PhysRevLett.104.238102 PMC4929007

[25] Weber SC, Thompson MA, Moerner WE, Spakowitz AJ, Theriot JA (2012) Analytical Tools To Distinguish the Effects of Localization Error, Confinement, and Medium Elasticity on the Velocity Autocorrelation Function. Biophys J 102: 2443–2450. 22713559 10.1016/j.bpj.2012.03.062 PMC3368140

[26] Polka JK, Kollman JM, Mullins RD (2014) Accessory factors promote AlfA-dependent plasmid segregation by regulating filament nucleation, disassembly, and bundling. Proc Natl Acad Sci USA 111: 2176–2181. 24481252 10.1073/pnas.1304127111 PMC3926056

[27] Sliusarenko O, Heinritz J, Emonet T, Jacobs-Wagner C (2011) High-throughput, subpixel precision analysis of bacterial morphogenesis and intracellular spatio-temporal dynamics. Mol Microbiol 80: 612–627. 21414037 10.1111/j.1365-2958.2011.07579.x PMC3090749

[28] Fisher JK, Bourniquel A, Witz G, Weiner B, Prentiss M, et al (2013) Four-Dimensional Imaging of E. coli Nucleoid Organization and Dynamics in Living Cells. Cell 153: 882–895. 23623305 10.1016/j.cell.2013.04.006 PMC3670778

[29] Wiggins PA, Cheveralls KC, Martin JS, Lintner R, Kondev J (2010) Strong intranucleoid interactions organize the Escherichia coli chromosome into a nucleoid filament. Proc Natl Acad Sci USA 107: 4991–4995. 20194778 10.1073/pnas.0912062107 PMC2841910

[30] Hadizadeh Yazdi N, Guet CC, Johnson RC, Marko JF (2012) Variation of the folding and dynamics of the Escherichia coli chromosome with growth conditions. Mol Microbiol 86: 1318–1333. 23078205 10.1111/mmi.12071 PMC3524407

[31] Swulius MT, Jensen GJ (2012) The Helical MreB Cytoskeleton in Escherichia coli MC1000/pLE7 Is an Artifact of the N-Terminal Yellow Fluorescent Protein Tag. J Bacteriol 194: 6382–6386. 22904287 10.1128/JB.00505-12 PMC3497537

[32] Badrinarayanan A, Lesterlin C, Reyes-Lamothe R, Sherratt D (2012) The Escherichia coli SMC Complex, MukBEF, Shapes Nucleoid Organization Independently of DNA Replication. J Bacteriol 194: 4669–4676. 22753058 10.1128/JB.00957-12 PMC3415497

[33] Mercier R, Petit MA, Schbath S, Robin S, El Karoui M, et al (2008) The MatP/matS Site-Specific System Organizes the Terminus Region of the E-coli Chromosome into a Macrodomain. Cell 135: 475–485. 18984159 10.1016/j.cell.2008.08.031

[34] Nollmann M, Crisona NJ, Arimondo PB (2007) Thirty years of Escherichia coli DNA gyrase: From in vivo function to single-molecule mechanism. Biochimie 89: 490–499. 17397985 10.1016/j.biochi.2007.02.012

[35] Ezaki B, Ogura T, Niki H, Hiraga S (1991) Partitioning of a Mini-F Plasmid into Anucleate Cells of the Mukb Null Mutant. J Bacteriol 173: 6643–6646. 1917886 10.1128/jb.173.20.6643-6646.1991 PMC209005

[36] Funnell BE, Gagnier L (1995) Partition of P1 Plasmids in Escherichia-Coli Mukb Chromosomal Partition Mutants. J Bacteriol 177: 2381–2386. 7730268 10.1128/jb.177.9.2381-2386.1995 PMC176895

[37] Tao WT, Dasgupta S, Nordstrom K (2000) Role of the mukB gene in chromosome and plasmid partition in Escherichia coli. Mol Microbiol 38: 392–400. 11069664 10.1046/j.1365-2958.2000.02138.x

[38] Derman AI, Lim-Fong G, Pogliano J (2008) Intracellular mobility of plasmid DNA is limited by the ParA family of partitioning systems. Mol Microbiol 67: 935–946. 18208495 10.1111/j.1365-2958.2007.06066.x

[39] Banigan EJ, Gelbart MA, Gitai Z, Wingreen NS, Liu AJ (2011) Filament depolymerization can explain chromosome pulling during bacterial mitosis. PLoS Comput Biol 7: e1002145. 21966261 10.1371/journal.pcbi.1002145 PMC3178632

[40] Lau IF, Filipe SR, Soballe B, Okstad OA, Barre FX, et al (2003) Spatial and temporal organization of replicating Escherichia coli chromosomes. Mol Microbiol 49: 731–743. 12864855 10.1046/j.1365-2958.2003.03640.x

[41] Thevenaz P, Ruttimann UE, Unser M (1998) A pyramid approach to subpixel registration based on intensity. Ieee T Image Process 7: 27–41. 10.1109/83.650848 18267377

[42] Manders EMM, Verbeek FJ, Aten JA (1993) Measurement of Colocalization of Objects in Dual-Color Confocal Images. J Microsc-Oxford 169: 375–382. 10.1111/j.1365-2818.1993.tb03313.x 33930978

