# Supplementary figures and images for: Competing ParA Structures Space Bacterial Plasmids Equally over the Nucleoid
Source: PLoS Comput Biol. 2014 Dec 18;10(12):e1004009. doi: 10.1371/journal.pcbi.1004009 (PMC4270457; doi:10.1371/journal.pcbi.1004009)

**S1.**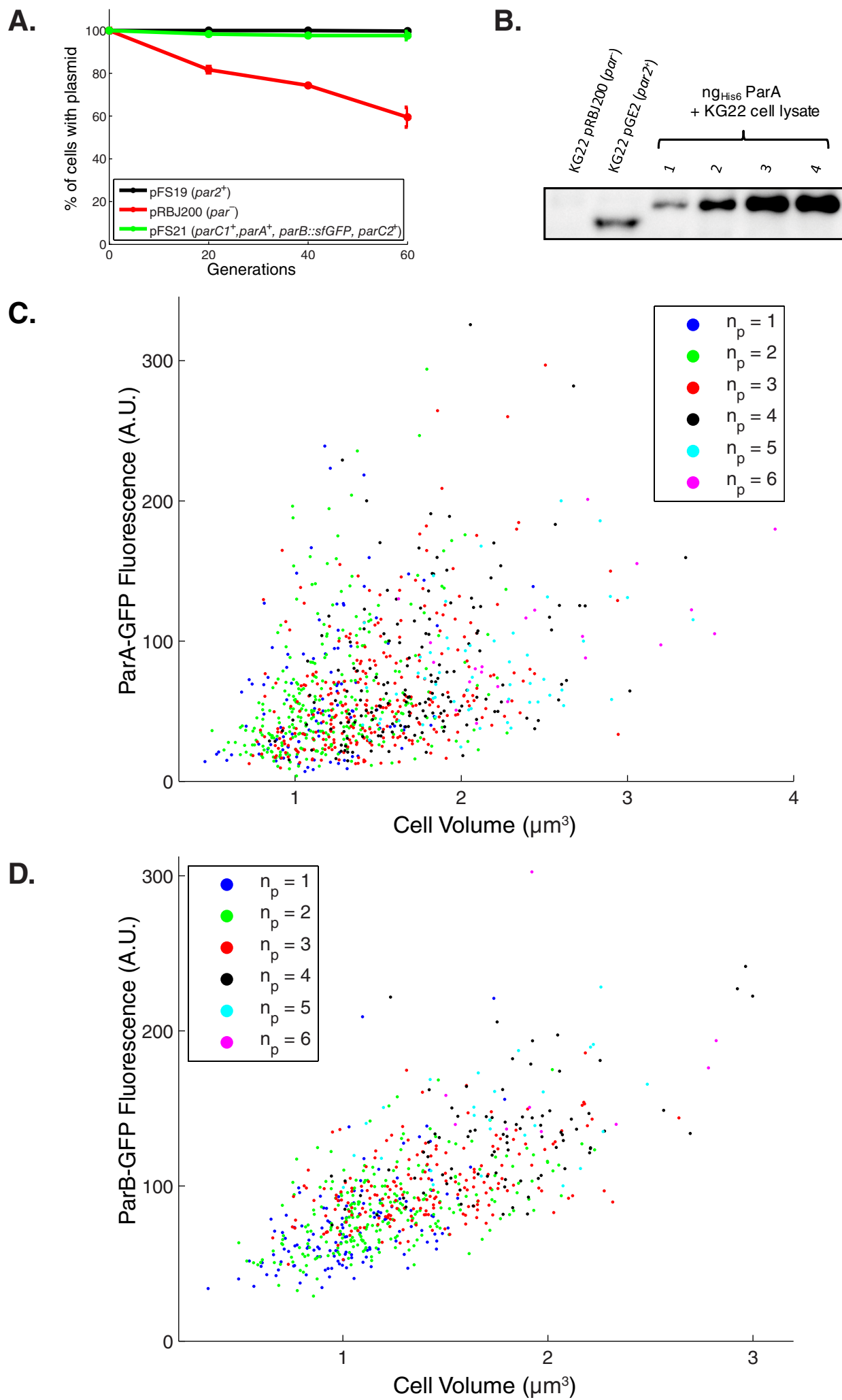

Supplement: S1 Fig — par2 protein functionality and expression levels. (A) Plasmid loss-frequency assay showing pFS21 stabilisation to wild-type levels by the recombinant par2 locus encoding parB::sfGFP, confirming functionality of the fluorescent fusion protein. Plasmids used are pRBJ200 (par -, red), pFS19 (par2 +, black) and experimental vector pFS21 (parC1+, parA+, parB::sfGFP, parC2 +, green), n = 2, error bars: standard error of the mean. (B) Representative section of semi-quantitative Western blot used for approximating ParA molecule numbers in vivo. Cell lysate samples of strain KG22 carrying a mini-R1 plasmid lacking (pRBJ200) or containing par2 (pGE2) were compared to plasmid-free KG22 cell lysate mixed with known amounts of purified His6ParA. Standard curve generated from intensity measurements from this blot has R2 = 0.965. Band intensities were measured and quantified using the ImageQuant TL 1D Gel Analysis Software, (n = 3). (C) Scatter plot of ParA-GFP total fluorescence signal in single WT cells as a function of cell volume, when expressed from an inducible promoter (Plac). The different color labels indicate the number of plasmid foci. Plasmids: pSR233 (mini-R1, par2+, Plac::parA::eGFP, tetO120) and pSR124 (PBAD::tetR::mCherry). (D) Scatter plot of ParB-GFP total fluorescence signal in single WT cells as a function of cell volume, when expressed from its native promoter. Plasmid: pFS21 (parC1+, parA+, parB::sfGFP, parC2 +); color labeling as in (C). (PDF) [file pcbi.1004009.s001.pdf]

**S2.****A.**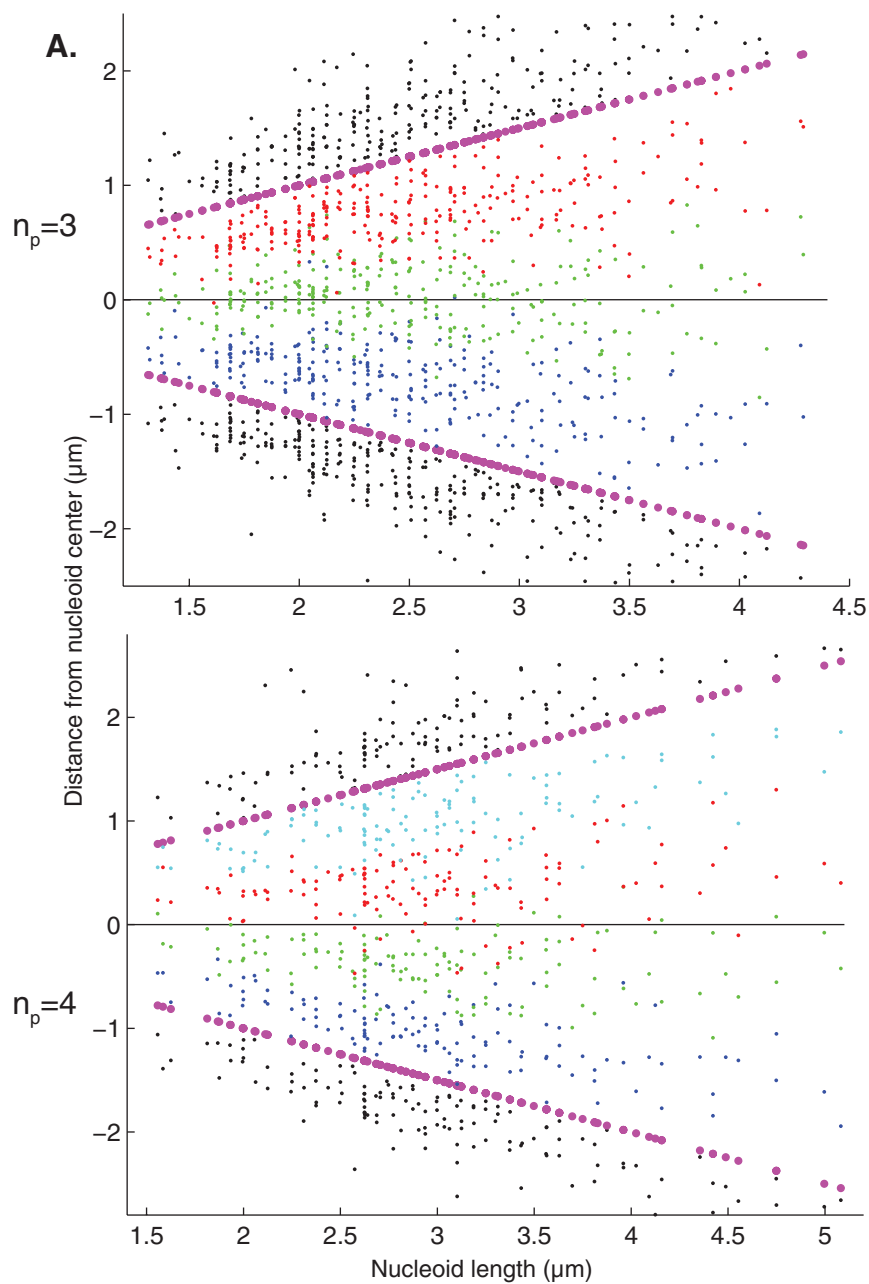**B.**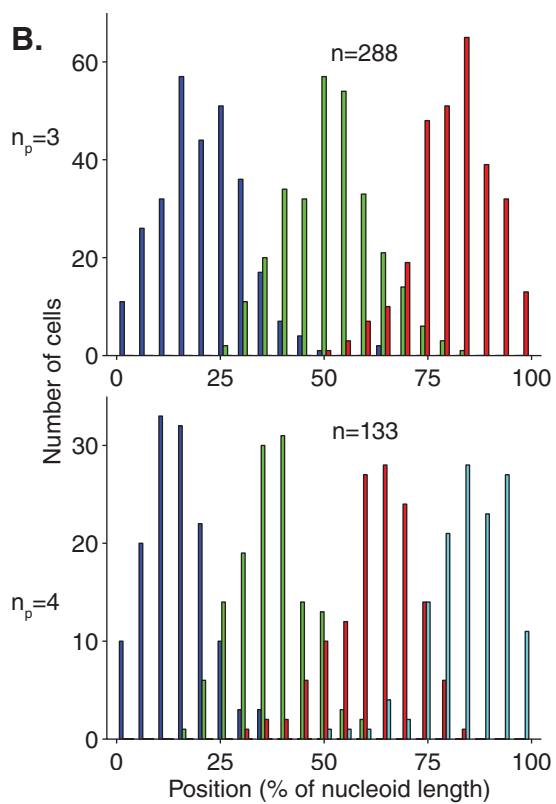

Supplement: S2 Fig — Plasmid foci are equally spaced over the nucleoid irrespective of nucleoid length or plasmid focus copy number. (A) Scatter plot of plasmid foci positions (blue, green, red, cyan) with respect to nucleoid edges (purple) and cell edges (black) for wild-type cells. Strains and plasmids used for S2 Fig. are as described in Fig. 1. (B) Histograms of plasmid foci positions shown in (A) relative to nucleoid length. (PDF) [file pcbi.1004009.s002.pdf]

S3.

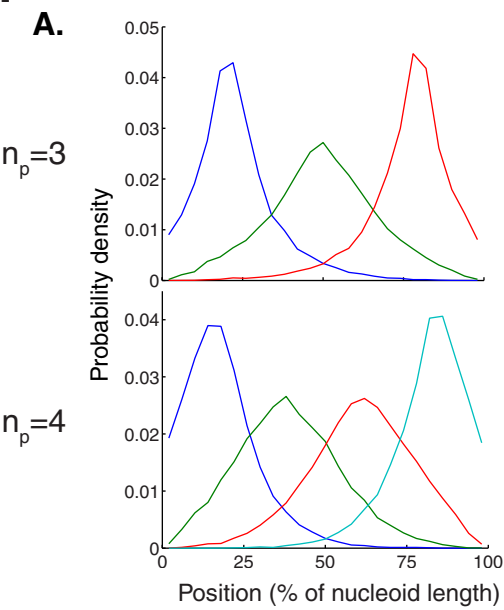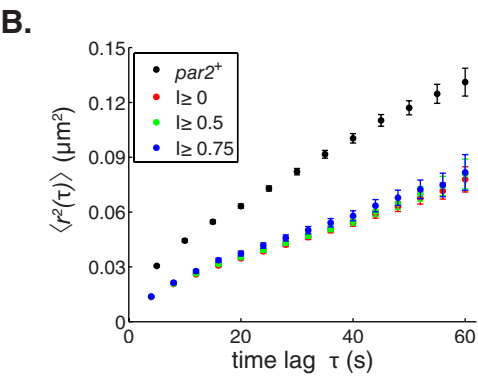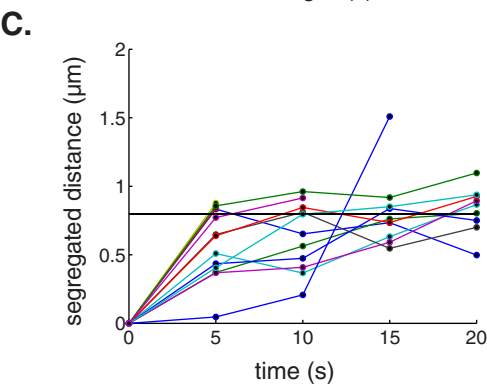

Supplement: S3 Fig — Diffusion/immobilization model can move and maintain plasmids at equally spaced positions. (A) Time-averaged plasmid position distributions for diffusion/immobilization model with np = 3,4 on a simulated nucleoid growing from 1.5 µm to 3 µm in 40 min without plasmid duplication. Plasmid distributions were obtained by sampling positions every 5 s in 36 independent simulations. (B) Plots as in Fig. 3B except with experimental par- (red, green, blue) plasmid trajectories in which plasmid location is within a region of normalized Hoechst stain intensity I equal to or higher than the values indicated in the legend. The corresponding plasmid copy numbers (npar2+ = 763, npar-, I≥0 = 747, npar-, I≥0.5 = 592, npar-, I≥0.75 = 401) indicate that a large fraction of par- plasmids do indeed reside in the nucleoid region; error bars: standard error of the mean. (C) Plots of 13 segregation events of par2+ pSR236 (mini-R1, parC1+, parA-, parB+, parC2+, tetO120, Plac::parA::eGFP) plasmids in E. coli cells harboring pSR124 (PBAD::tetR::mCherry). Shown is the additionally segregated distance (colored lines) as a function of time, both with respect to the start of each segregation event. A segregation event is defined as two foci that are initially ≤0.3 µm apart and subsequently segregate ≥0.8 µm further apart within 20 s. The horizontal line (black) indicates 0.8 µm. (PDF) [file pcbi.1004009.s003.pdf]

**S4.****A.**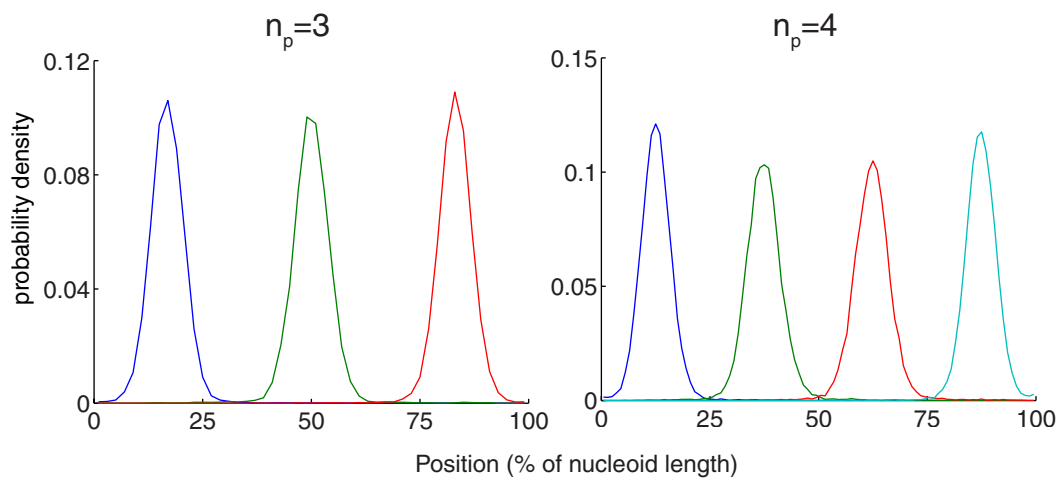**B.**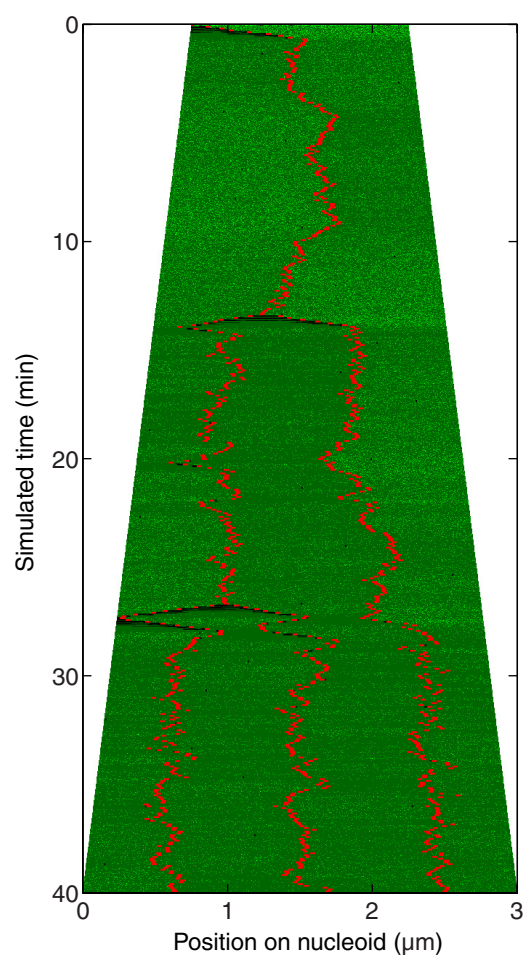**C.**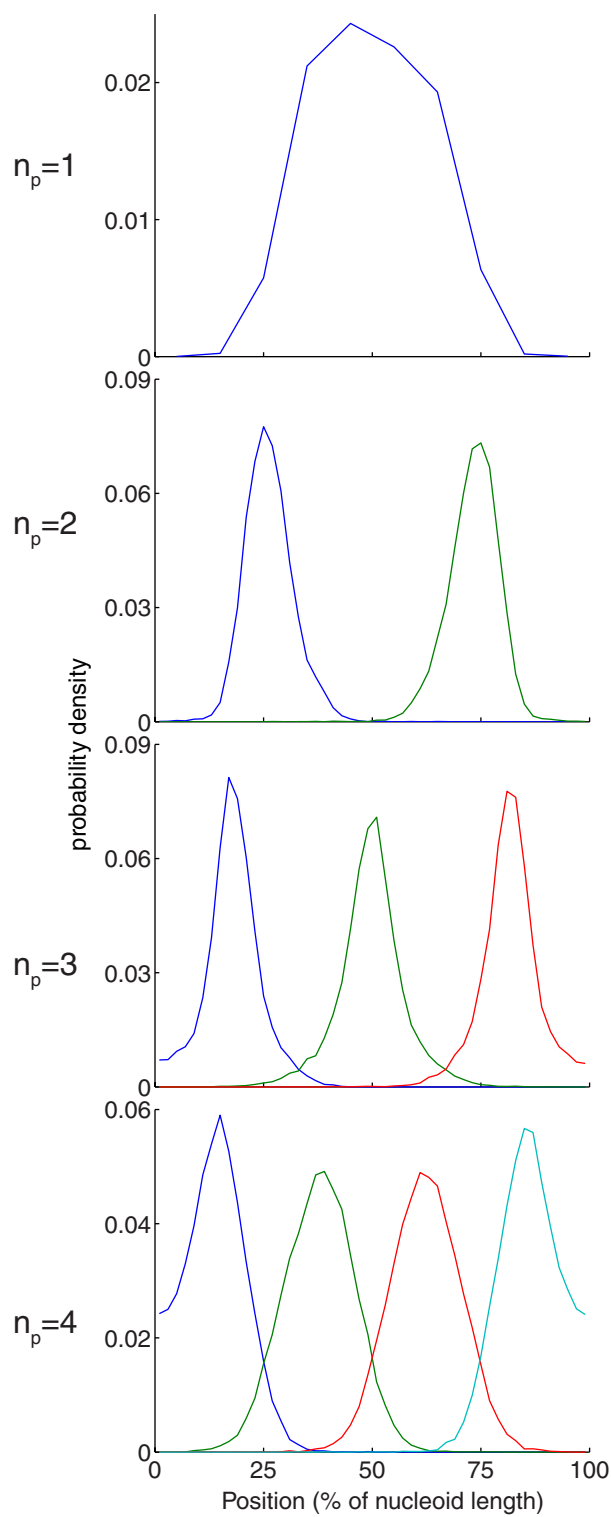

Supplement: S4 Fig — The directed motion model can equally space plasmids over the nucleoid, and is not critically dependent on the extent of ParA polymerization. (A) Time-averaged plasmid position distributions for directed motion model with short polymers with np = 3,4 plasmids on a simulated nucleoid growing from 1.5 µm to 3 µm in 40 min without plasmid duplication. Plasmid distributions were obtained by sampling positions every 5 s in 36 independent simulations. (B) Typical simulation kymograph of the directed motion model with long polymers. Long polymers extend from nucleoid ends in a growing cell, where plasmid (red) is initially directed from a nucleoid edge to mid-cell by ParA (green) filament competition. After plasmid duplication, the system dynamically self-organizes to reacquire equal plasmid spacing. (C) Time-averaged plasmid position distributions for directed motion model with long polymers with np = 1–4 plasmids. Simulated nucleoid growth and plasmid distributions obtained as in (A). (PDF) [file pcbi.1004009.s004.pdf]

## S5.

A.

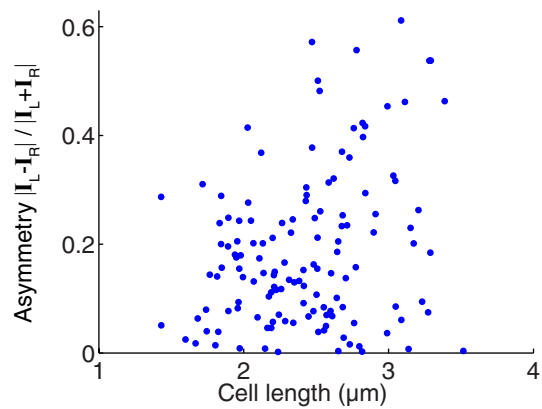

B.

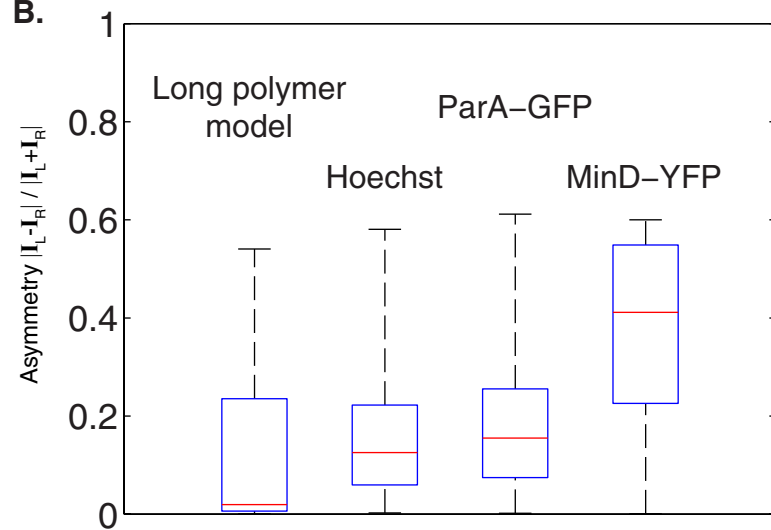

C.

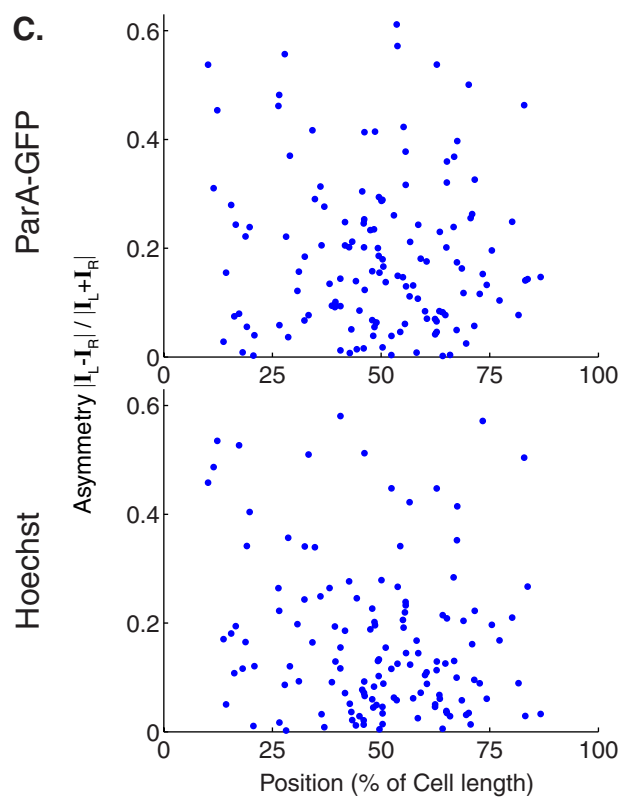

Supplement: S5 Fig — Hoechst DNA stain and ParA-GFP signal asymmetry are relatively low and uncorrelated to plasmid focus positioning. (A) Scatter plot of ParA-GFP asymmetry measure as a function of cell length (n = 134). (B) ParA asymmetry prediction from the directed motion model with long polymers. Comparison shown to experimental ParA-GFP (n = 134), Hoechst (n = 134) and MinD-YFP distributions [7]. (C) Scatter plot of ParA-GFP and Hoechst asymmetry as a function of (a single) plasmid focus position relative to cell length. (PDF) [file pcbi.1004009.s005.pdf]

**S6.****A.****WT****Nal**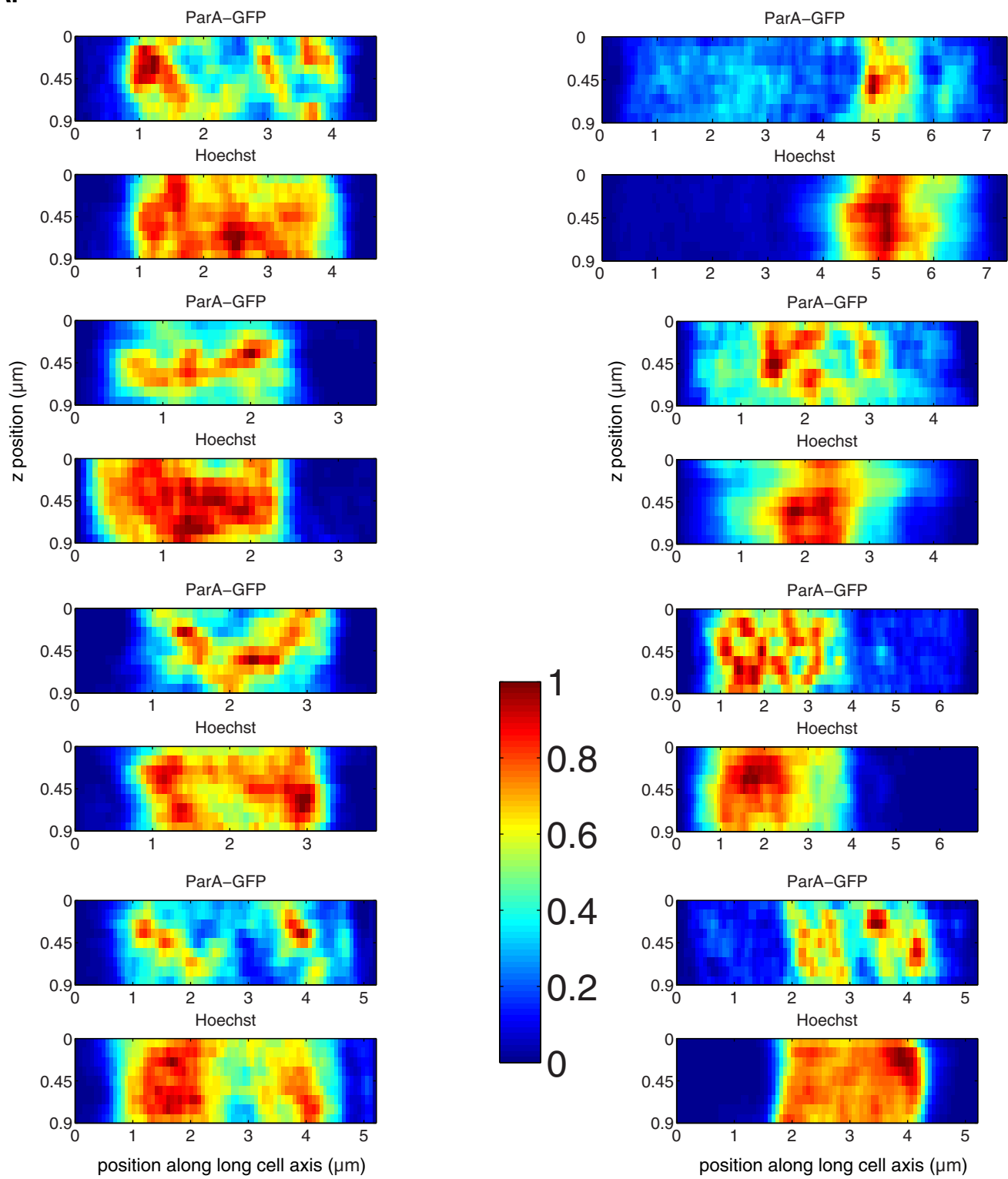**B.****WT****Nal**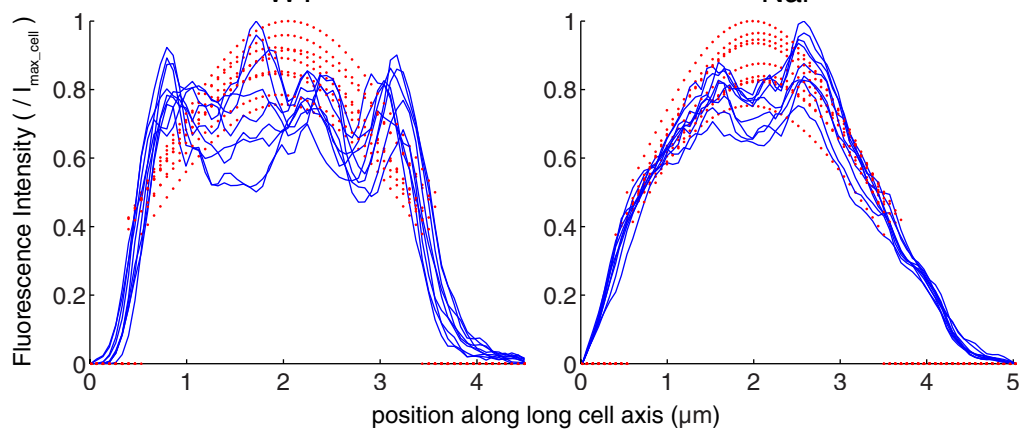

Supplement: S6 Fig — Correlation between Hoechst and ParA-GFP distributions. (A) Normalized fluorescence intensity profiles along the long cell axis for 9 in focus z heights (dz = 0.1 µm) resulting from deconvolved Z-stacks in representative WT and Nal-treated strains. Many cases (representative examples shown) support the existence of linear ParA-GFP structures, although the inherent optical resolution of the imaging prohibits stronger conclusions about the presence or absence of narrow linear ParA-GFP filaments. For every cell having detectable Hoechst and ParA-GFP signals, the corresponding profiles were used for the systematic colocalization analyses. (B) Graphical illustration of the unbiased systematic ‘first harmonic’ analysis of deconvolved 3d Hoechst signal inside representative cells in WT and Nal-treated strains. The Hoechst (blue) profiles indicate the signal intensities (integrated over the cell width) along the long cell axis at 9 in focus z heights with corresponding ‘first harmonics’ (dotted red curves, see Materials and Methods). Fluorescence signal distributions deviate significantly more from the first harmonics in WT compared to Nal-treated cells (Wilcoxon rank sum test, p<10−149), showing that Hoechst DNA stain distributions are perturbed in the latter. This analysis is independent of nucleoid length, which is altered in Nal-treated strains as compared to WT (S7A Fig.). (PDF) [file pcbi.1004009.s006.pdf]
