# Supplementary material for: Competing ParA Structures Space Bacterial Plasmids Equally over the Nucleoid
Source: PLoS Comput Biol. 2014 Dec 18;10(12):e1004009. doi: 10.1371/journal.pcbi.1004009 (PMC4270457; doi:10.1371/journal.pcbi.1004009)
Supplement: S7 Fig — Comparison of plasmid foci position histograms in cells with perturbed nucleoid morphology to completely randomized plasmid distributions. (A) Mean nucleoid length (error bars: standard error of the mean) of cells used for the plasmid positioning analysis shown in Figs. 1B, S2A and (B,C) in different strains: WT (n = 1695), mukE (n = 1378), mukF (n = 1555), matP mutants (n = 2995) and cells treated with nalidixic acid (Nal) (n = 1127). According to unpaired t tests, all mutants and Nal show a mean differing from WT (p<10−3). Although the average nucleoid length in matP mutants decreased, the average number of nucleoids per cell increased compared to WT (p<10−41) due to a large fraction of cells exhibiting 2 nucleoids (using our half maximum criteria). This observation is consistent with the previously proposed function of MatP in preventing early segregation of duplicated Ter macrodomains. (B) Scatter plot of np = 1–4 plasmid foci positions (blue, green, red, cyan) with respect to nucleoid edges (purple) and cell edges (black) for mukE, mukF mutant cells. (C) As in (B) for matP mutants and cells treated with 50 µg/ml nalidixic acid (Nal). (D) Histograms of np = 3,4 plasmid foci positions shown in (B,C) relative to nucleoid size. (E) Histograms of 105 datasets for each of np = 1–4, where for each dataset plasmids are positioned in [0,100] with a uniform distribution, independent from each other and consequently labeled 1..np according to their position. This protocol induces an inherent spatial ordering. By comparing these distributions with the WT experimental data shown in Fig. 1C (np = 1,2) and S2B Fig. (np = 3,4) it is clear that the parABC system positions plasmid foci much more precisely, although the effect of active positioning becomes less clear as np increases. (F) Time-averaged plasmid position distributions for directed motion model with short and long polymers for np = 3–4 (short) and np = 1–4 (long) on simulated growing nucleoids without plasmid duplica [file pcbi.1004009.s007.pdf]

S7.

A.

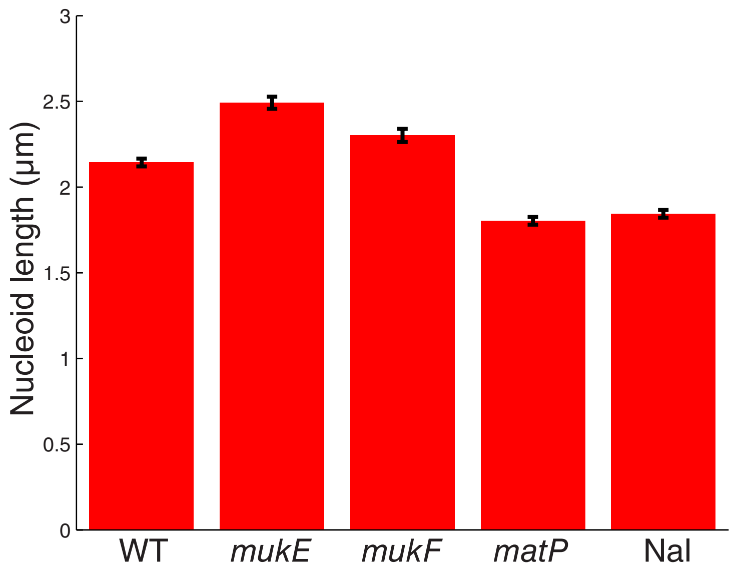

S7.

B.

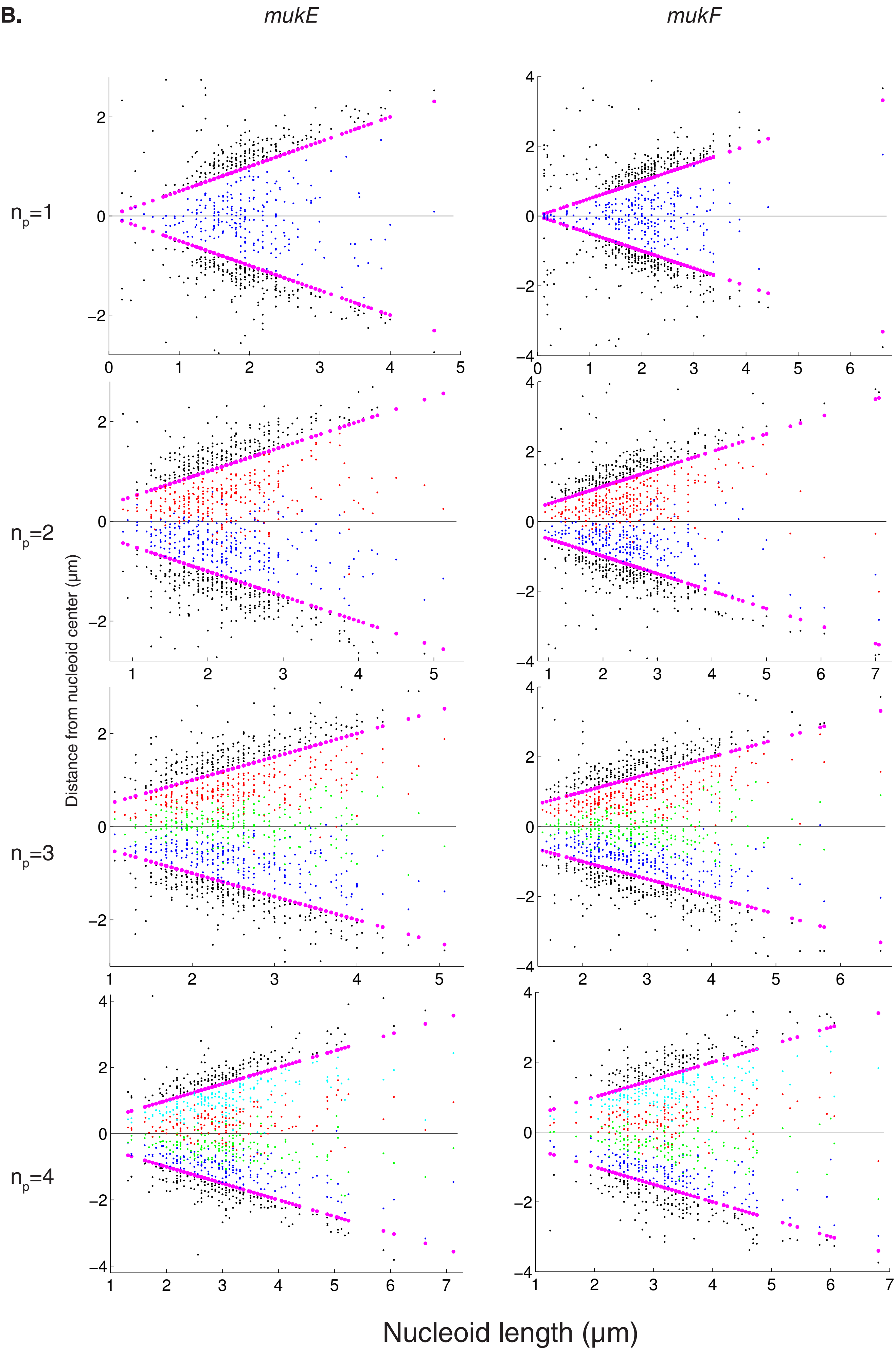

mukE
mukF
n_p=1
n_p=2
n_p=3
n_p=4
Distance from nucleoid center (μm)
Nucleoid length (μm)

S7.
c.

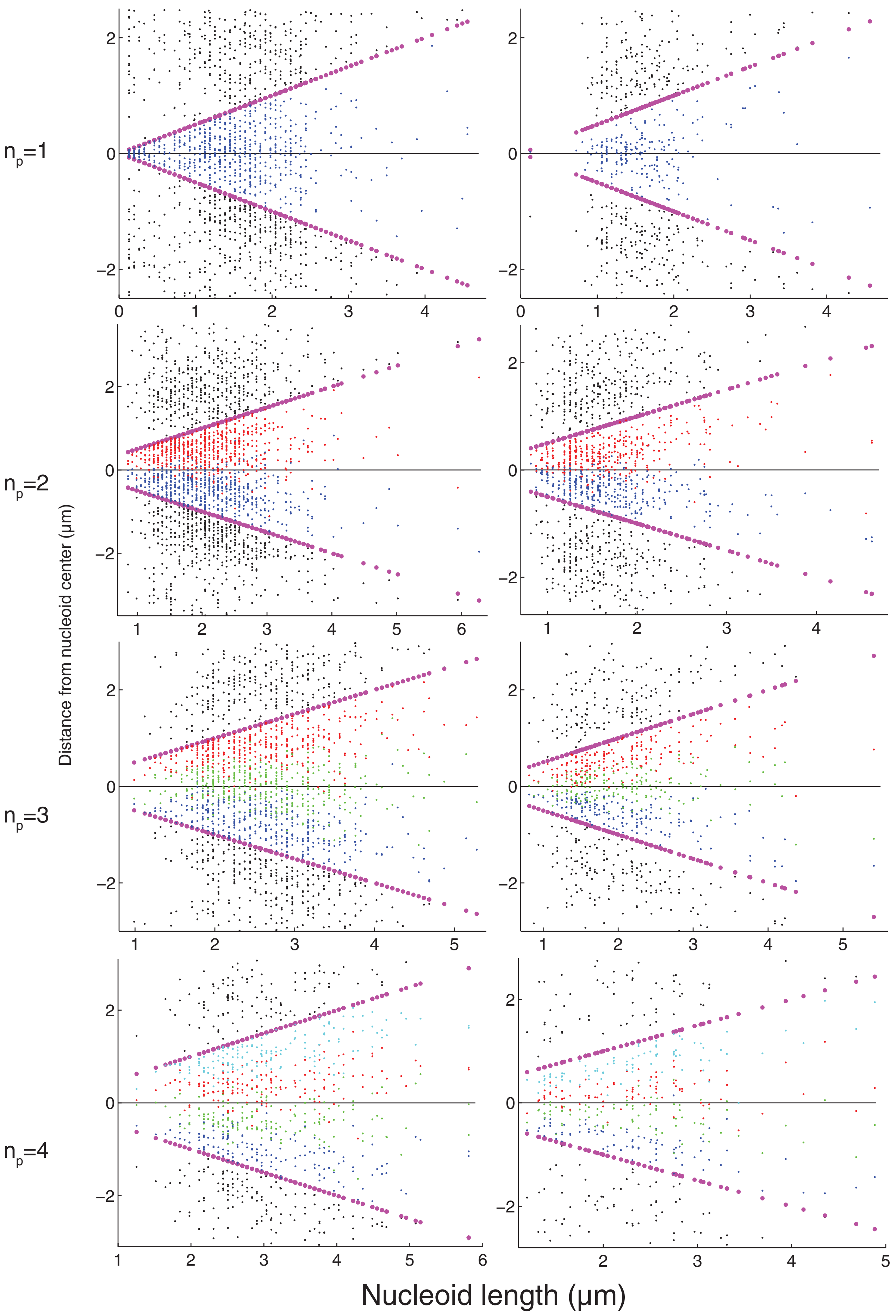
matP
Nal
n_p=1
n_p=2
n_p=3
n_p=4
Distance from nucleoid center (µm)
Nucleoid length (µm)

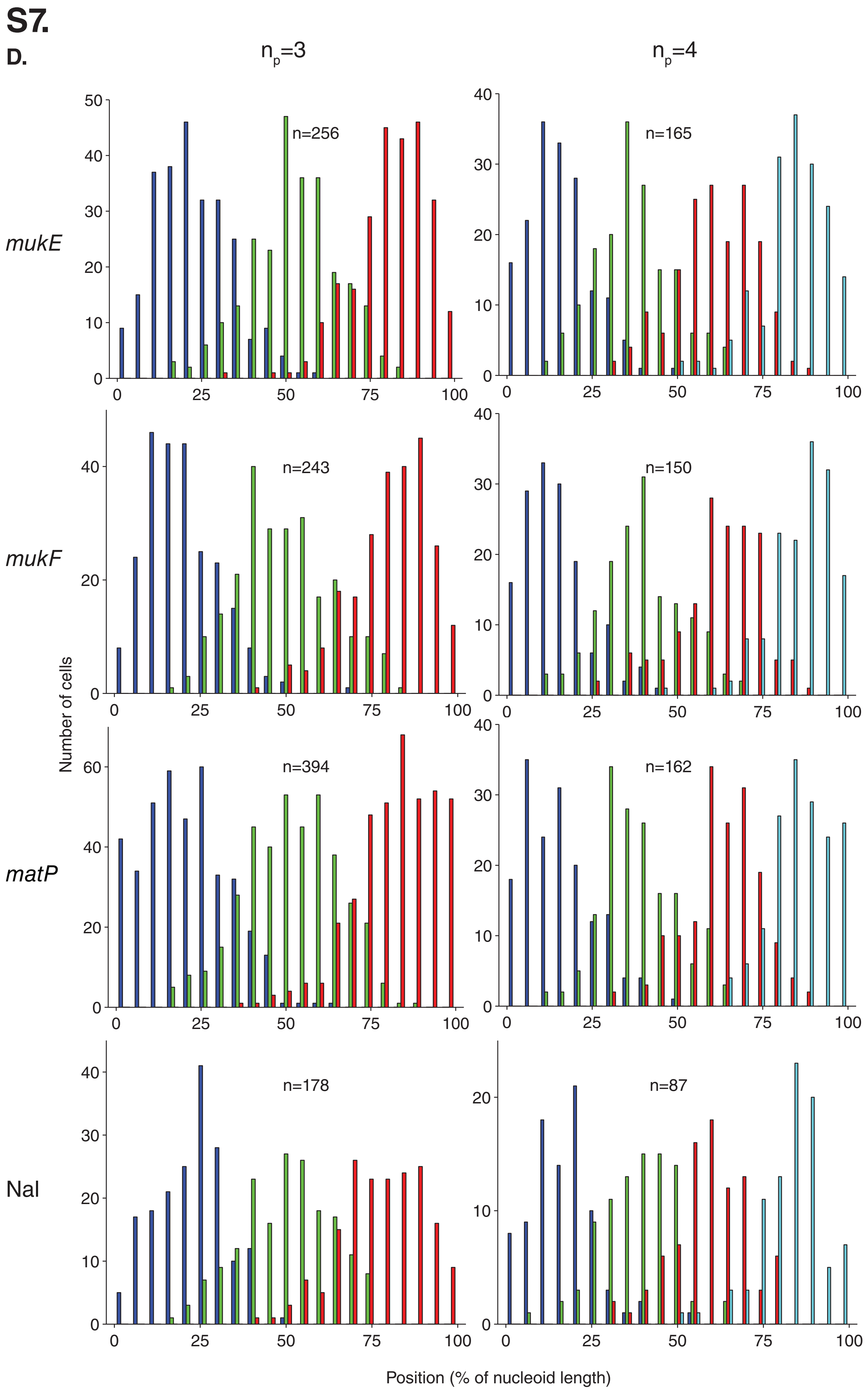
S7.
D.
n_p=3
n_p=4
mukE
mukF
matP
Nal
n=256
n=165
n=243
n=150
n=394
n=162
n=178
n=87
Number of cells
Position (% of nucleoid length)

S7.

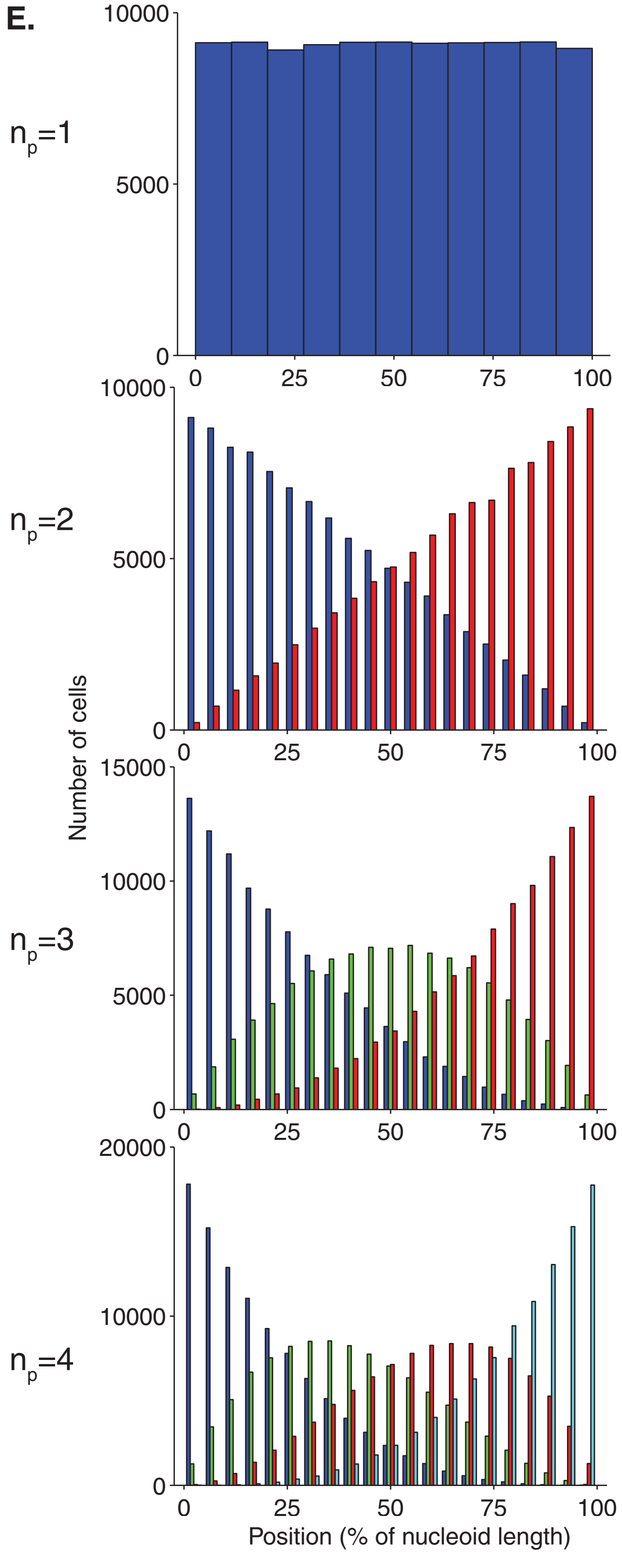

E.
np=1
np=2
np=3
np=4
10000
5000
0
0
25
50
75
100
15000
20000
Number of cells
Position (% of nucleoid length)

S7.

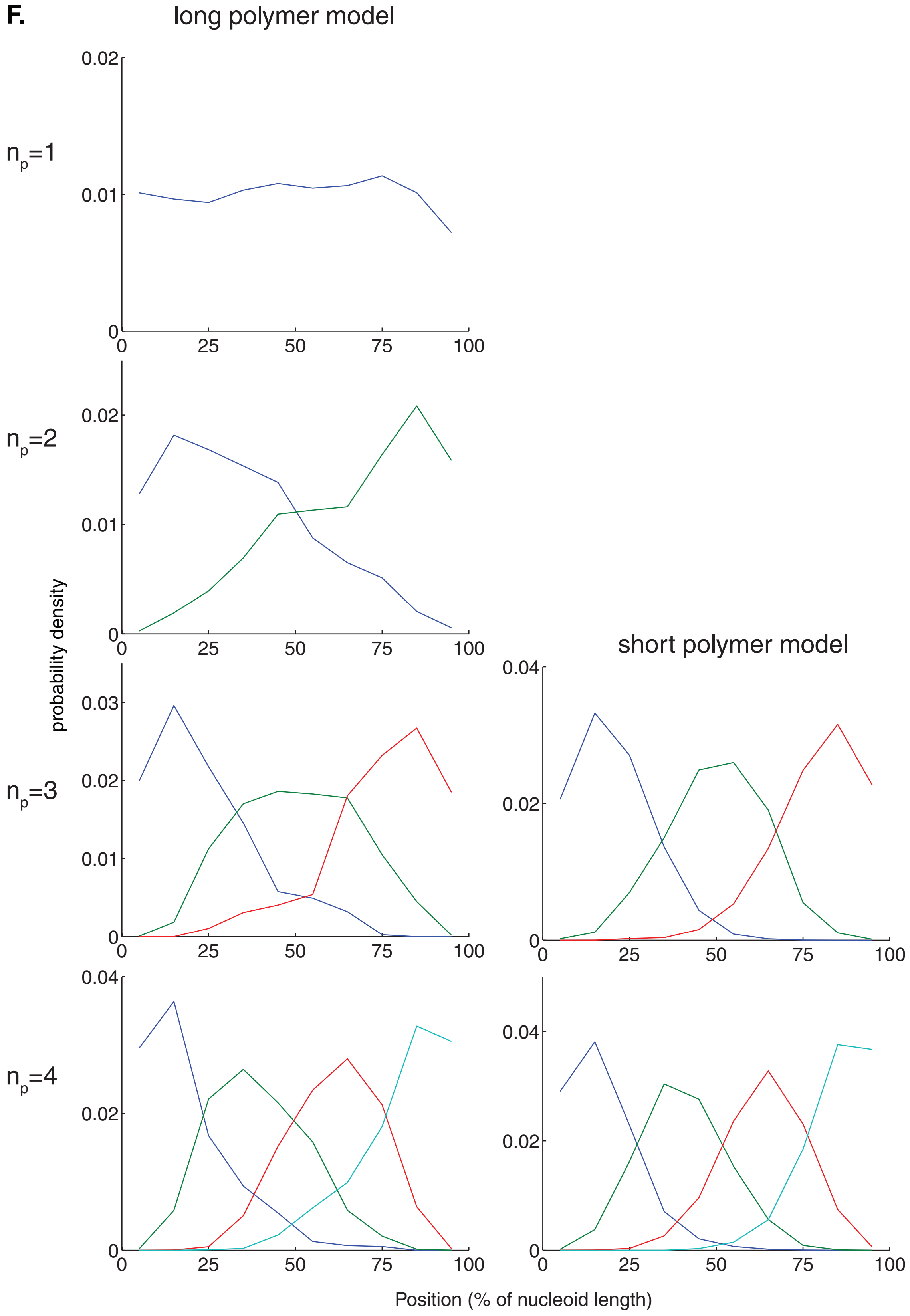
F.
long polymer model
short polymer model
n_p=1
n_p=2
n_p=3
n_p=4
probability density
0.02
0.01
0
0.03
0.04
0
25
50
75
100
Position (% of nucleoid length)
