# Supplementary material for: Competing ParA Structures Space Bacterial Plasmids Equally over the Nucleoid
Source: PLoS Comput Biol. 2014 Dec 18;10(12):e1004009. doi: 10.1371/journal.pcbi.1004009 (PMC4270457; doi:10.1371/journal.pcbi.1004009)
Supplement: S1 Text — Supplementary text to section: Mathematical analysis shows that dynamic ParA concentrations can generate equal plasmid spacing. This text contains the derivation that in our mathematical model a symmetric ParA concentration implies equal plasmid spacing in case of ParB-parC-mediated ParA-ATP hydrolysis with any rate kB. (PDF) [file pcbi.1004009.s008.pdf]

Supplementary Text to section: Mathematical analysis shows that dynamic ParA concentrations can generate equal plasmid spacing.

1 Expression for $\mathrm{A}(x)$

In the main text we described how ParA symmetry results in equal plasmid spacing. In there we assumed that the ParA concentration at each plasmid is zero in order to gain understanding of the mechanism and keep the analysis concise. Here we show that equal plasmid spacing can also be achieved in case the ParA concentration at each plasmid is not zero, a more realistic scenario. In fact all that is required is the less stringent condition that ParB-*parC* complexes mediate ParA turnover at the plasmid.

We model the nucleoid as a 1d system of length L (along the cell long axis) on which ParA-ATP and plasmids can interact. $\mathrm{A}(x,t)$ denotes the nucleoid-associated ParA-ATP concentration at position x relative to one nucleoid edge at time t and the cytoplasmic ParA copy number. Let $x_1(t)..x_{n_p}(t)$ be the positions of the n_p plasmids. At each plasmid, ParA-ATP can be hydrolyzed with rate k_B, turning Par-ATP into a cytoplasmic ParA form, with copy number $\mathrm{A_c}(t)$. After sufficiently long timescales, the cytoplasmic ParA becomes competent to bind the nucleoid again, with rate $\mathrm{J}(\mathrm{A_c}(t))$. Once bound to the nucleoid, ParA-ATP can diffuse along the nucleoid with diffusion constant D. This system can be described by the deterministic reaction-diffusion equations:

$$\frac{\partial \mathrm{A}(x,t)}{\partial t} = D\frac{\partial^2 \mathrm{A}(x,t)}{\partial x^2} - k_B\sum_{i=1}^{n_p}\mathrm{A}(x_i(t))\delta\left(x - x_i(t)\right) + \frac{\mathrm{J}(\mathrm{A_c}(t))}{L}$$

$$\frac{\mathrm{dA_c}(t)}{\mathrm{d}t} = k_B\sum_{i=1}^{n_p}\mathrm{A}(x_i(t)) - \mathrm{J}(\mathrm{A_c}(t)) \qquad (1)$$

$$\text{Boundary Conditions}: \left.\frac{\partial \mathrm{A}(x,t)}{\partial x}\right|_{x=0} = 0 = \left.\frac{\partial \mathrm{A}(x,t)}{\partial x}\right|_{x=L} \text{ for all t.}$$

Here $\delta(x)$ indicates the Dirac delta function. In line with the experimental evidence we assume that cytoplasmic ParA binds to the nucleoid with a slow rate k_W after becoming cytoplasmic: $\mathrm{J}(\mathrm{A_c}(t)) = k_W\mathrm{A_c}(t)$. Assuming a given (time-independent) total ParA copy number $\mathrm{A_T}$ in the system, we will find that J is

determined implicitly: $\mathrm{A_T} = \mathrm{A_c}(t) + \int_0^L \mathrm{A}(\mathrm{J}(\mathrm{A_c(t)}), x, t)dx$. We will use this relation to calculate the steady state $\left(\frac{\partial \mathrm{A}(x,t)}{\partial t} = 0 = \frac{\mathrm{dA_c}(t)}{\mathrm{d}t}\right)$ solution $\mathrm{A}(x)$ for any given plasmid configuration $x_1...x_{n_p}$ with plasmid copy number $n_p = 1, 2$ in terms of $\mathrm{A_T}$ and the other parameters L, D, k_B and k_W. Note that the dimensions of k_B are length/time due to the dimensionality of the Dirac delta function $\delta(x)$. The procedure described here generalizes to any n_p.

Recall from the main text that by invoking a separation of timescales between ParA diffusion and plasmid motion, Eq. 1 reduces in steady state to:

$$\begin{aligned} &D\frac{\mathrm{d}^2\mathrm{A}(x)}{\mathrm{d}x^2} = k_B \sum_{i=1}^{n_p} \mathrm{A}(x_i)\delta\left(x - x_i\right) - \frac{\mathrm{J}}{L} \\ &\text{Boundary Conditions}: \left.\frac{\mathrm{dA}(x)}{\mathrm{d}x}\right|_{x=0} = 0 = \left.\frac{\mathrm{dA}(x)}{\mathrm{d}x}\right|_{x=L} \\ &k_W \mathrm{A_c} = \mathrm{J} = k_B \sum_{i=1}^{n_p} \mathrm{A}(x_i) \text{ (flux balance.)} \end{aligned} \tag{2}$$

Eq. 2 can be solved for $\mathrm{A}(x)$ in terms of J and $\mathrm{A}(x_1)...\mathrm{A}(x_{n_p})$ by using the Neumann boundary conditions:

$$\mathrm{A}(x) = \begin{cases} \frac{\mathrm{J}}{2LD}\left(x_1^2 - x^2\right) + \mathrm{A}(x_1) & \text{if } 0 \le x \le x_1, \\ \frac{k_B}{D}\sum_{i=1}^{j} \mathrm{A}(x_i)\left(x - x_j\right) + \frac{\mathrm{J}}{2LD}\left(x_j^2 - x^2\right) + \mathrm{A}(x_j) & \text{if } x_j \le x \le x_{j+1}, 1 \le j < n_p, \\ \frac{\mathrm{J}}{2LD}\left((L - x_{n_p})^2 - (L - x)^2\right) + \mathrm{A}(x_{n_p}) & \text{if } x_{n_p} \le x \le L. \end{cases} \tag{3}$$

Note that in order to be physically relevant we assume our solution $\mathrm{A}(x)$ to be a continuous function of x. This generates the following recursive relations between the concentrations at the plasmids:

$$\mathrm{A}(x_j) = \frac{k_B}{D}\sum_{i=1}^{j-1} \mathrm{A}(x_i)\left(x_j - x_{j-1}\right) + \frac{\mathrm{J}}{2LD}\left(x_{j-1}^2 - x_j^2\right) + \mathrm{A}(x_{j-1}) \text{ for } 1 < j \le n_p. \tag{4}$$

Now we focus on obtaining $\mathrm{A}(x_1)$ in the case $n_p = 1$ for any plasmid position x_1. Integration over the nucleoid results in $\int_0^L \mathrm{A}(x)dx = \mathrm{A}(x_1)L + \frac{\mathrm{J}}{D}\left[\frac{L^2}{3} - x_1 L + x_1^2\right]$. From conservation of total ParA in the system, J is determined implicitly in terms of $A(x_1)$: $\mathrm{J} = k_W \mathrm{A_c} = k_W\left(\mathrm{A_T} - \left[\mathrm{A}(x_1)L + \frac{J}{D}\left[\frac{L^2}{3} - x_1 L + x_1^2\right]\right]\right)$. Solving for J results in: $\mathrm{J} = \frac{\mathrm{A_T} - \mathrm{A}(x_1)L}{\frac{1}{k_W} + \frac{1}{D}\left[\frac{L^2}{3} - x_1 L + x_1^2\right]}$. Now we use the flux balance

constraint in Eq. 2 to obtain the desired $\mathrm{A}(x_1)$:

$$\text{for } n_p = 1\text{: } \mathrm{A}(x_1) = \frac{\mathrm{A_T}}{L + \frac{k_B}{k_W} + \frac{k_B}{D}\left[\frac{L^2}{3} - x_1 L + x_1^2\right]}.$$

Now that J and $\mathrm{A}(x_1)$ are obtained, the ParA distribution on the nucleoid as described by Eq. 3 is fully determined. For $n_p > 1$ this procedure can be repeated to find $\mathrm{A}(x_1)$. Due to the continuity constraint, Eq. 4, all the other $\mathrm{A}(x_j)$ are then also determined. We illustrate this for $n_p = 2$: piecewise integration over the nucleoid results in: $\int_0^L \mathrm{A}(x)dx = \mathrm{A}(x_1)L + \frac{\mathrm{J}}{D}\left(\frac{2}{3}L^2 + \frac{3}{2}x_2^2 - 2Lx_2 - \frac{1}{3L}x_2^3 + \frac{1}{2}x_1^2\right) + \frac{k_B}{2D}\mathrm{A}(x_1)\left(x_2 - x_1\right)\left(2L - x_1 - x_2\right)$. Now we use the conservation of total ParA again to obtain J in terms of $\mathrm{A}(x_1)$: $\mathrm{J} = \frac{\mathrm{A_T} - \mathrm{A}(x_1)L - \frac{k_B}{2D}\mathrm{A}(x_1)(x_2 - x_1)(2L - x_1 - x_2)}{\frac{1}{k_W} + \frac{1}{D}\left[\frac{2}{3}L^2 + \frac{3}{2}x_2^2 - 2Lx_2 - \frac{1}{3L}x_2^3 + \frac{1}{2}x_1^2\right]}$. Again turning to the flux balance condition, whilst realizing that by Eq. 4 $\mathrm{A}(x_2)$ is known in terms of $\mathrm{A}(x_1)$ and J we find: $\mathrm{J} = k_B\left[\mathrm{A}(x_1) + \frac{k_B}{D}\mathrm{A}(x_1)\left(x_2 - x_1\right) + \frac{\mathrm{J}}{2LD}\left(x_1^2 - x_2^2\right) + \mathrm{A}(x_1)\right]$. Now we solve for $\mathrm{A}(x_1)$:

$$\mathrm{A}(x_1) = \frac{\mathrm{A_T}}{L + \frac{k_B}{2D}\left(x_2 - x_1\right)\left(2L - x_1 - x_2\right) + \left[2k_B + \frac{k_B^2}{D}\left(x_2 - x_1\right)\right]\mathrm{B}},$$

with $\mathrm{B} = \frac{\frac{1}{k_W} + \frac{1}{D}\left[\frac{2}{3}L^2 + \frac{3}{2}x_2^2 - 2Lx_2 - \frac{1}{3L}x_2^3 + \frac{1}{2}x_1^2\right]}{1 + \frac{k_B}{2LD}\left(x_2^2 - x_1^2\right)}$.
This determines $\mathrm{A}(x)$ for $n_p = 2$ completely.

2 Derivation that ParA symmetry implies equal plasmid spacing

In this section we derive that for any $n_p \geq 1$ for our steady state solution $\mathrm{A}(x)$, the following statement holds:

$$\text{for all } j \text{ and } x \text{ such that } x_{j-1} \leq x_j - x \leq x_j \text{ and } x_j \leq x_j + x \leq x_{j+1}:$$
$$\mathrm{A}(x_j - x) = \mathrm{A}(x_j + x) \Longrightarrow \text{for all } j : x_j = \frac{L}{2n_p} + \frac{L}{n_p}(j-1)$$

Here it is understood that $1 \leq j \leq n_p$, indicating the label of the jth plasmid that are assumed to be ordered (without loss of generality): $x_1 \leq .. \leq x_{n_p}$. Furthermore we define $x_0 = 0$ and $x_{n_p+1} = L$. To show this we use the expressions for $\mathrm{A}(x)$ described in Eq. 3. First we focus on $j = n_p$. Let

$x_{n_p-1} \le x_{n_p} - x \le x_{n_p}$ and $x_{n_p} \le x_{n_p} + x \le L$ and $\mathrm{A}(x_{n_p} - x) = \mathrm{A}(x_{n_p} + x)$:

$$\begin{aligned}
&\frac{k_B}{D}\sum_{i=1}^{n_p-1}\mathrm{A}(x_i)\left((x_{n_p}-x)-x_{n_p-1}\right)+\frac{\mathrm{J}}{2LD}\left(x_{n_p-1}^2-(x_{n_p}-x)^2\right)+\mathrm{A}(x_{n_p-1}) = \\
&\qquad \frac{\mathrm{J}}{2LD}\left((L-x_{n_p})^2-(L-(x_{n_p}+x))^2\right)+\mathrm{A}(x_{n_p}) \Rightarrow \\
&\qquad \left(\frac{k_B}{D}\sum_{i=1}^{n_p-1}\mathrm{A}(x_i)+\frac{\mathrm{J}}{D}\right)x = \frac{2J}{LD}x_{n_p}x \text{ for all x} \Rightarrow \\
&\qquad x_{n_p} = \frac{L}{2}\left(1+\frac{k_B}{\mathrm{J}}\sum_{i=1}^{n_p-1}\mathrm{A}(x_i)\right).
\end{aligned} \tag{5}$$

Note that we used the continuity requirement (4) here for $\mathrm{A}(x_{n_p})$ on the right hand side. In the special case of $n_p = 1$, the left hand side of the equation can be replaced by $\frac{\mathrm{J}}{2LD}\left(x_1^2 - (x_1 - x)^2\right) + \mathrm{A}(x_1)$, which using the same procedure leads straightforwardly to the desired result $x_1 = \frac{L}{2}$. Now in the case of $n_p > 1$, we proceed with $j = 1$. Let $0 \le x_1 - x \le x_1$ and $x_1 \le x_1 + x \le x_2$ and $\mathrm{A}(x_1 - x) = \mathrm{A}(x_1 + x)$:

$$\begin{aligned}
&\frac{\mathrm{J}}{2LD}\left(x_1^2-(x_1-x)^2\right)+\mathrm{A}(x_1) = \\
&\qquad \frac{k_B}{D}\mathrm{A}(x_1)\left((x_1+x)-x_1\right)+\frac{\mathrm{J}}{2LD}\left(x_1^2-(x_1+x)^2\right)+\mathrm{A}(x_1) \Rightarrow \\
&\qquad 2\frac{\mathrm{J}}{L}x_1x = k_B\mathrm{A}(x_1)x \text{ for all x} \Rightarrow \\
&\qquad x_1 = \frac{Lk_B}{2\mathrm{J}}\mathrm{A}(x_1).
\end{aligned}$$

Proceeding with $1 < j < n_p$, we let $x_{j-1} \le x_j - x \le x_j$ and $x_j \le x_j + x \le x_{j+1}$ and use again Eq. 4 to replace $\mathrm{A}(x_j)$:

$$\begin{aligned}
&\frac{k_B}{D}\sum_{i=1}^{j-1}\mathrm{A}(x_i)\left((x_j-x)-x_{j-1}\right)+\frac{\mathrm{J}}{2LD}\left(x_{j-1}^2-(x_j-x)^2\right)+\mathrm{A}(x_{j-1}) = \\
&\frac{k_B}{D}\sum_{i=1}^{j}\mathrm{A}(x_i)\left((x_j+x)-x_j\right)+\frac{\mathrm{J}}{2LD}\left(x_j^2-(x_j+x)^2\right)+\mathrm{A}(x_j) \Rightarrow \\
&\frac{\mathrm{J}}{2L}(4x_jx) = k_B\left[2\sum_{i=1}^{j-1}\mathrm{A}(x_i)+\mathrm{A}(x_j)\right]x \text{ for all x} \Rightarrow \\
&x_j = \frac{Lk_B}{\mathrm{J}}\sum_{i=1}^{j-1}\mathrm{A}(x_i)+\frac{Lk_B}{2\mathrm{J}}\mathrm{A}(x_j).
\end{aligned}$$

Now we have direct relations between the concentrations at each plasmid and the position of the plasmids for symmetric concentrations. Note that by math-

ematical induction, it is straightforward to show that for $1 \leq j < n_p$:

$$\mathrm{A}(x_j) = \frac{2\mathrm{J}}{Lk_B}\left(x_j + 2\sum_{i=1}^{j-1}(-1)^{j-i}x_i\right). \tag{6}$$

Now we define the plasmid spacings $z_j := x_j - x_{j-1}$. Note that by Eq. 6 for $1 < j < n_p$: $z_j = \frac{Lk_B}{2J}\left(\mathrm{A}(x_j) + \mathrm{A}(x_{j-1})\right)$. We can then replace all $\mathrm{A}(x_i)$ in Eq. 4 in terms of the x_j and z_j and subsequently solve for the spacing and positions. First we rewrite Eq. 4 as:

$$\mathrm{A}(x_j) + \mathrm{A}(x_{j-1}) = \frac{k_B}{D}\sum_{i=1}^{j-1}\mathrm{A}(x_i)z_j - \frac{J}{2LD}z_j\left(z_j + 2x_{j-1}\right) + 2\mathrm{A}(x_{j-1}) \Rightarrow$$

$$\frac{2}{k_B}z_j = \begin{cases} \frac{2}{D}\left(\sum_{m=1}^{\frac{j}{2}-1} z_{2m+1} + x_1\right)z_j - \frac{1}{2D}z_j\left(z_j + 2x_{j-1}\right) + \frac{4}{k_B}\left(x_{j-1} + 2\sum_{i=1}^{j-2}(-1)^{j-1-i}x_i\right) & \text{if j even} \\ \frac{2}{D}\sum_{m=1}^{\frac{j-1}{2}} z_{2m}z_j - \frac{1}{2D}z_j\left(z_j + 2x_{j-1}\right) + \frac{4}{k_B}\left(x_{j-1} + 2\sum_{i=1}^{j-2}(-1)^{j-1-i}x_i\right) & \text{if j odd} \end{cases}$$

These equations are essentially quadratic equations in z_j. Using mathematical induction we will now show that $z_j = 2x_1$ for all $2 \leq j < n_p$. We start with the base case $j = 2$: $z_2\left[\frac{2}{k_B} - \frac{2}{D}x_1 + \frac{1}{2D}(z_2 + 2x_1)\right] = \frac{4}{k_B}x_1$. The only physical solution is indeed $z_2 = 2x_1$. Now assume that the induction hypothesis holds true for all i such that $2 \leq i < j$. Then for j odd:

$$z_j\left[\frac{2}{k_B} - \frac{2}{D}\sum_{m=1}^{\frac{j-1}{2}} z_{2m} + \frac{1}{2D}(z_j + 2x_{j-1})\right] = \frac{4}{k_B}\left(x_{j-1} + 2\sum_{i=1}^{j-2}(-1)^{j-1-i}x_i\right) \Rightarrow$$

$$z_j\left[\frac{2}{k_B} - \frac{2}{D}\frac{j-1}{2}2x_1 + \frac{1}{2D}(z_j + 2(2(j-1)-1)x_1)\right] =$$

$$\frac{4}{k_B}\left((2(j-1)-1)x_1 + 2\sum_{i=1}^{j-2}(-1)^{j-1-i}(2i-1)x_1\right) \Rightarrow$$

$$z_j = 2x_1$$

For j even, the same procedure also results in $z_j = 2x_1$. This concludes the induction argument. Now we have for $1 \leq j < n_p : x_j = (2j-1)x_1$. Lastly we focus on $j = n_p$: first note that we can now simplify Eq. 5 to: $x_{n_p} = \frac{L}{2} + (n_p - 1)x_1$. This means that $z_{n_p} = x_{n_p} - x_{n_p-1} = \frac{L}{2} + (2 - n_p)x_1$. Finally, by flux balance (Eq. 2): $k_B\mathrm{A}(x_{n_p}) = \mathrm{J} - k_B\sum_{i=1}^{n_p-1}\mathrm{A}(x_i)$. Invoking continuity (Eq. 4) for $\mathrm{A}(x_{n_p})$ and replacing $\sum_{i=1}^{n_p-1}\mathrm{A}(x_i)$ for plasmid spacings, we obtain:

$$\frac{L}{k_B} = \begin{cases} \left[\frac{2}{k_B} + \frac{2}{D}z_{n_p}\right]\left[\sum_{m=1}^{\frac{n_p}{2}-1} z_{2m+1} + x_1\right] - \frac{1}{2D}z_{n_p}\left(z_{n_p} + 2x_{n_p-1}\right) + \frac{2}{k_B}x_1 & \text{if } n_p \text{ even} \\ \left[\frac{2}{k_B} + \frac{2}{D}z_{n_p}\right]\sum_{m=1}^{\frac{n_p-1}{2}} z_{2m} - \frac{1}{2D}z_{n_p}\left(z_{n_p} + 2x_{n_p-1}\right) + \frac{2}{k_B}x_1 & \text{if } n_p \text{ odd} \end{cases}$$

$$\Rightarrow \frac{L}{k_B} = \left[\frac{2}{k_B} + \frac{2}{D}z_{n_p}\right](n_p - 1)x_1 - \frac{1}{2D}z_{n_p}\left(z_{n_p} + 2(2(n_p-1)-1)x_1\right) + \frac{2}{k_B}x_1$$

Now we insert our expression $z_{n_p} = \frac{L}{2} + (2 - n_p)x_1$ to find that $x_1 = \frac{L}{2n_p}$. We conclude that a symmetric ParA concentration leads to equal plasmid spacing: $1 \leq j \leq n_p : x_j = \frac{L}{2n_p} + (j-1)\frac{L}{n_p}$.
