# Supplementary material for: Competing ParA Structures Space Bacterial Plasmids Equally over the Nucleoid
Source: PLoS Comput Biol. 2014 Dec 18;10(12):e1004009. doi: 10.1371/journal.pcbi.1004009 (PMC4270457; doi:10.1371/journal.pcbi.1004009)
Supplement: S2 Text — Supplementary materials and methods including Table S1, S2 and S3. (DOCX) [file pcbi.1004009.s009.docx]

**Supplementary Text 2: supplementary materials and methods section**

*Plasmid construction*

Plasmid pFS21 was constructed by fusing *parB* to the N-terminus of the *sfGFP* gene [1]. The resulting fusion encoded a 5 amino acid linker (Ile-Pro-Leu-Glu-Leu) between the last C-terminal residue of ParB and the first N-terminal residue of sfGFP. The multiple cloning site (MCS) of pGE2 (mini-R1, *par2*^+^) downstream of *par2* was extended through the insertion of aligned oligonucleotides FS29 (TCGAGTCTAGAGCGGCCGCGGTACCG) and FS30 (TCGACGGTACCGCGGCCGCTCTAGAC) to provide additional restriction enzyme recognition sites, resulting in plasmid pFS19. The *parB::sfGFP* fusion gene was inserted into *par2* to replace wild-type *parB* by digestion of both vector and fragment with AscI/XbaI and subsequent ligation of the DNA fragments, creating plasmid pFS20 (mini-R1, *parC1*^+^, *parA*^+^, *parB::sfGFP*, *parC2^-^*). The *parC2* region lost in this step was restored by digestion of the recombinant vector with NotI/XbaI and ligation with a similarly digested PCR fragment containing *parC2*, yielding the final construct, plasmid pFS21 (mini-R1, *parC1*^+^, *parA*^+^, *parB::sfGFP*, *parC2*^+^). The *parC2* fragment was generated using PCR and primers FS35 (CCCCCTCTAGATTAAAAACACTAACGTAATAATCAATAAGTTATTACTTA) and FS36 (CCCCCGCGGCCGCAATGCTGAAAAACAGACACGCATAATTCATA) using pGE2 as a template. The final construct was confirmed by sequencing using DNA Sequencing & Services (MRCPPU, College of Life Sciences, University of Dundee, Scotland, [www.dnaseq.co.uk](http://www.dnaseq.co.uk)). The modified *par2* locus encoding the ParB-sfGFP fusion protein was subsequently tested for functionality using plasmid loss-frequency assays (according to the method published in [2]). The recombinant *par2* locus was found to be fully functional and to stabilize pFS21 at wild-type *par2* levels, as measured using pFS19 (see Fig. S1A).

*Strain construction*

*E. coli* strain FS1 was constructed using P1 transduction, with BW25113 *matP::kan* [3] serving as donor and wild-type KG22 as recipient strain. The kanamycin resistance cassette is flanked by FLP recombinase recognition sequences, which were used to excise the resistance cassette by transforming the KG22 *matP::kan* strain with plasmid pCP20 and expressing the FLP recombinase according to the published method [4]. The loss of the resistance cassette was confirmed by PCR using a specific oligonucleotide primer pair: matP-up2 (CAGCACACGTAGA) and matP-down2 (GCGTAGAGATCG) [both kindly provided by Dr. Elisa Galli]. The resulting strain was named FS1 (KG22Δ*matP*).

*E. coli* strains FS2 and FS3 were generated by transducing the recipient wild-type KG22 with P1 lysates obtained from strains AZ5450 (*mukE::kan*) or AZ5381 (*mukF::kan*), respectively [5]. Both strains were verified by PCR using the primers mukE-F (CAAGCAGATTTCACCGGACT) and mukE-R (GATTTTCAATCGGCATTGCT) (FS2) or mukF-F (AGCTGGTTGGCAAATTATGG) and mukF-R (AGTCCGGTGAAATCTGCTTG) (FS3).

*Semi-quantitative Western blots for approximating the number of ParA molecules per cell* in vivo*.*

Stationary phase cultures of *E. coli* strain KG22 harbouring either: pRBJ200 (*par^-^*), pGE2 (*par2^+^*) or a no plasmid control were back-diluted to an OD_450_ = 0.025 in supplemented M9 medium without antibiotics. Cultures were incubated while being shaken at 37^o^C for approximately 3 ½ h. After reaching an OD_450_ = 0.4 samples were taken for SDS-PAGE and CFU (colony-forming units) determination. Cells were pelleted by centrifugation at 1800g at 4^o^C. After careful removal of the supernatant, pellets were re-suspended in a 50:50 mix of PBS and 2 x Laemmli buffer. Protein preparations were immediately denatured at 100^o^C for 10 minutes. Known amounts of a purified _His6_ParA preparation were mixed with plasmid-free KG22 cell lysate to serve as concentration standards. Proteins were run on a 6-15% SDS-PAGE gel at 180 V for approximately 3 ½ h to fully resolve the bands in the ≈24 kDa range. Separated proteins in the gel were transferred onto PVDF membranes (GE Healthcare) using a wet transfer method and wet transfer cassette (Bio-Rad) with a PowerPac HC set to a current of 0.35 A for 60 minutes. ParA protein was detected using affinity-purified polyclonal rabbit _His6_ParA antibody (diluted 1:1,000) and detected with a goat anti-rabbit-IgG horseradish peroxidase-conjugated secondary antibody (1:10,000; Sigma) and Pierce ECL 2 Western Blotting Substrate (Thermo Scientific). The resulting chemiluminescence was detected using the ImageQuant LAS4000 mini system (GE Healthcare). Band intensities of the known _His6_ParA concentration standards were used to generate a standard curve and estimate the protein content of the wild-type ParA in KG22 pGE2 cell lysate samples. Typical standard curves generated from this method were highly significant (r^2^ > 0.95). The number of ParA molecules per cell was calculated after determining the CFU per millilitre of cell culture after spreading culture samples from a ten-fold dilution series on NA plates containing and lacking antibiotic.

*Total ParA fluorescence analysis*

Using the same data set as for the ParA asymmetry analysis, we also computed the total ParA-GFP intensity in every cell by summing over the linear projection values along the long axis. The cell volume (in pixels^3^) was determined by MicrobeTracker. We then multiplied this by the pixel size cubed to compute the total volume. Results are shown in Fig. S1C.

*Total ParB fluorescence analysis*

Using the same data set (consisting of single confocal planes) as for the determination of plasmid foci positioning in WT cells, we computed the total ParB-GFP intensity per cell as described in the previous section. Results are shown in Fig. S1D and are similar for summed ParB-GFP Z-stacks.

*Previous polymer model is not consistent with observed ParA and ParB levels*

We also investigated mechanistic implementations of an alternative polymer model previously proposed in [6]. There, a filament length-dependent plasmid fall off-rate resulted in equal plasmid spacing. In cases where the ParB or ParA levels are tightly controlled and scale with the plasmid copy number n­_p_, but not with cell volume, such a length dependent off-rate could in principle be achieved. However, in Fig. S1D, we show that ParB-GFP signal intensity, expressed from its native promoter, rather scales with cell volume, irrespective of n_p_. ParA-GFP, when expressed from an inducible promoter, exhibits a notably more varying expression level (Fig. S1C), again without scaling with n_p_. Such inducible control does not, however, affect the plasmid positioning dynamics [6]. Both these observations are inconsistent with alternative polymer models that require careful coordination of ParA and ParB levels with plasmid copy number.

*Random plasmid positioning histograms*

For a given plasmid copy number n_p_ ranging from 1 to 4, we sampled a set of n_p_ independent plasmid positions from a one dimensional uniform distribution on [0,100]. Each plasmid position set was ordered from small to large. We repeated this process 10^5^ times and subsequently generated histograms of the ordered plasmid positions (Fig. S7E). Note that the experimental plasmid focus positioning histograms are significantly more ordered than these (compare Fig. 1C, S2B). Additionally, the expected interplasmid distance for randomly positioned plasmid pairs (n_p_=2) is 1/3 of the nucleoid length, while the observed spacing is 0.5 (Fig. 1D). These results demonstrate the effectiveness of the *parABC* plasmid positioning system.

**Table S1.** Strains used and constructed in this study.

| **Name** | **Genotype** | **Resistance** | **Source** |
| --- | --- | --- | --- |
| KG22 | C600 *(supE44, rpsL, rfbD1, thi1, leuB6, lacY1, tonA21): lacI^q^, lacZ*Δ*M1* | - | Laboratory collection |
| SR1 | KG22: Δ*pcnB* | Cat | [6] |
| BW25113Δ*matP::kan* | F-, Δ*(araD-araB)567*, Δ*lacZ4787*(*::rrnB-3*), *λ-*, *rph-1*, Δ*(rhaD-rhaB)568*, *hsdR514*, Δ*matP::kan* | Kan | [3] |
| AZ5450 | *mukE::kan* | Kan | [5] |
| AZ5381 | *mukF::kan* | Kan | [5] |
| FS1 | KG22: Δ*matP* | - | This work |
| FS2 | KG22: *mukE::kan* | Kan | This work |
| FS3 | KG22: *mukF::kan* | Kan | This work |

**Table S2.** Plasmids used and constructed in this study.

| **Name** | **Genotype / Notes** | **Resistance** | **Replicon** | **Source** |
| --- | --- | --- | --- | --- |
| pCP20 | *FLP^+^,* λ *cI857^+^,* λ *p_R_ Rep^ts^* | Amp, Cat | pUC | [4] |
| pGE220 | *P_lac_::parA::eGFP* | Amp | pUC | [8] |
| pRBJ200 | *par^-^* | Amp | R1^ts^ | Laboratory collection |
| pGE2 | *par2^+^* | Amp | R1^ts^ | [8] |
| pGE230 | *par^-^, P_lac_::parA::eGFP* | Kan | R1^ts^ | [8] |
| pSR124 | *P_BAD_::tetR::mCherry* | Amp | pMB1 | [6] |
| pSR233 | *par2^+^*, *P_lac_::parA::eGFP*, *tetO120* | Kan | R1^ts^ | [6] |
| pSR236 | *parC1^+^, ΔparA, parB^+^, parC2^+^, P_lac_::parA::eGFP, tetO120* | Kan | R1^ts^ | [6] |
| pMH82tetO120 | *par^-^*, *tetO120* | Kan | R1^ts^ | [6] |
| pFS19 | pGE2 with extended MCS | Amp | R1^ts^ | This work |
| pFS20 | *parC1^+^*, *parA^+^*, *parB::sfGFP*, *parC2*^-^ | Amp | R1^ts^ | This work |
| pFS21 | *parC1^+^*, *parA^+^*, *parB::sfGFP*, *parC2*^+^ | Amp | R1^ts^ | This work |

**Table S3.** Filter set specifics for (A) Olympus IX71 and (B) Zeiss Axiovert 200M microscopes. Wavelengths are given in nm. All filter cubes manufactured by Chroma.

|  | **Filter** | **Excitation wavelength** | **Emission wavelength** |
| --- | --- | --- | --- |
| **A** | **DAPI** | 360/40 | 457/50 |
|  | **FITC** | 490/20 | 528/38 |
|  | **RD-TR-Cy3** | 555/28 | 617/73 |
| **B** | **DAPI** | 350/50 | 460/50 |
|  | **ET-GFP** | 470/40 | 525/50 |
|  | **ET-mCherry** | 560/40 | 630/75 |

**Supplemental References**

1. Pedelacq JD, Cabantous S, Tran T, Terwilliger TC, Waldo GS (2006) Engineering and characterization of a superfolder green fluorescent protein. Nat Biotechnol 24: 79-88.

2. Gerdes K, Molin S (1986) Partitioning of Plasmid-R1 - Structural and Functional-Analysis of the Para Locus. J Mol Biol 190: 269-279.

3. Baba T, Ara T, Hasegawa M, Takai Y, Okumura Y, et al. (2006) Construction of Escherichia coli K-12 in-frame, single-gene knockout mutants: the Keio collection. Mol Syst Biol 2: 2006.0008.

4. Cherepanov PP, Wackernagel W (1995) Gene disruption in Escherichia coli: TcR and KmR cassettes with the option of Flp-catalyzed excision of the antibiotic-resistance determinant. Gene 158: 9-14.

5. Yamanaka K, Ogura T, Niki H, Hiraga S (1996) Identification of two new genes, mukE and mukF, involved in chromosome partitioning in Escherichia coli. Mol Gen Genet 250: 241-251.

6. Ringgaard S, van Zon J, Howard M, Gerdes K (2009) Movement and equipositioning of plasmids by ParA filament disassembly. Proceedings of the National Academy of Sciences of the United States of America 106: 19369-19374.

7. Sliusarenko O, Heinritz J, Emonet T, Jacobs-Wagner C (2011) High-throughput, subpixel precision analysis of bacterial morphogenesis and intracellular spatio-temporal dynamics. Mol Microbiol 80: 612-627.

8. Ebersbach G, Gerdes K (2001) The double par locus of virulence factor pB171: DNA segregation is correlated with oscillation of ParA. Proceedings of the National Academy of Sciences of the United States of America 98: 15078-15083.
